# Supplementary material for: Music intervention for sleep quality in critically ill and surgical patients: a meta-analysis
Source: BMJ Open. 2021 May 10;11(5):e042510. doi: 10.1136/bmjopen-2020-042510 (PMC8112429; doi:10.1136/bmjopen-2020-042510)
Supplement: Supplementary data [file bmjopen-2020-042510supp001.pdf]

Supplementary file 1 PRISMA checklist

Section/topic	#	Checklist item	Reported on page #
**TITLE**			
Title	1	Identify the report as a systematic review, meta-analysis, or both.	1
**ABSTRACT**			
Structured summary	2	Provide a structured summary including, as applicable: background; objectives; data sources; study eligibility criteria, participants, and interventions; study appraisal and synthesis methods; results; limitations; conclusions and implications of key findings; systematic review registration number.	2+3
**INTRODUCTION**			
Rationale	3	Describe the rationale for the review in the context of what is already known.	4
Objectives	4	Provide an explicit statement of questions being addressed with reference to participants, interventions, comparisons, outcomes, and study design (PICOS).	5
**METHODS**			
Protocol and registration	5	Indicate if a review protocol exists, if and where it can be accessed (e.g., Web address), and, if available, provide registration information including registration number.	7
Eligibility criteria	6	Specify study characteristics (e.g., PICOS, length of follow-up) and report characteristics (e.g., years considered, language, publication status) used as criteria for eligibility, giving rationale.	7+8
Information sources	7	Describe all information sources (e.g., databases with dates of coverage, contact with study authors to identify additional studies) in the search and date last searched.	7
Search	8	Present full electronic search strategy for at least one database, including any limits used, such that it could be repeated.	Supplementary file 2
Study selection	9	State the process for selecting studies (i.e., screening, eligibility, included in systematic review, and, if applicable, included in the meta-analysis).	7+8
Data collection process	10	Describe method of data extraction from reports (e.g., piloted forms, independently, in duplicate) and any processes for obtaining and confirming data from investigators.	8
Data items	11	List and define all variables for which data were sought (e.g., PICOS, funding sources) and any assumptions and simplifications made.	8

Risk of bias in individual studies	12	Describe methods used for assessing risk of bias of individual studies (including specification of whether this was done at the study or outcome level), and how this information is to be used in any data synthesis.	9
Summary measures	13	State the principal summary measures (e.g., risk ratio, difference in means).	9
Synthesis of results	14	Describe the methods of handling data and combining results of studies, if done, including measures of consistency (e.g., I^2) for each meta-analysis.	9
Risk of bias across studies	15	Specify any assessment of risk of bias that may affect the cumulative evidence (e.g., publication bias, selective reporting within studies).	9
Additional analyses	16	Describe methods of additional analyses (e.g., sensitivity or subgroup analyses, meta-regression), if done, indicating which were pre-specified.	NA
**RESULTS**			
Study selection	17	Give numbers of studies screened, assessed for eligibility, and included in the review, with reasons for exclusions at each stage, ideally with a flow diagram.	11
Study characteristics	18	For each study, present characteristics for which data were extracted (e.g., study size, PICOS, follow-up period) and provide the citations.	11-15, Tables 1 and 2
Risk of bias within studies	19	Present data on risk of bias of each study and, if available, any outcome level assessment (see item 12).	17
Results of individual studies	20	For all outcomes considered (benefits or harms), present, for each study: (a) simple summary data for each intervention group (b) effect estimates and confidence intervals, ideally with a forest plot.	Figures 2A, B, and C
Synthesis of results	21	Present results of each meta-analysis done, including confidence intervals and measures of consistency.	15-17
Risk of bias across studies	22	Present results of any assessment of risk of bias across studies (see Item 15).	17+18
Additional analysis	23	Give results of additional analyses, if done (e.g., sensitivity or subgroup analyses, meta-regression [see Item 16]).	NA
**DISCUSSION**			
Summary of evidence	24	Summarize the main findings including the strength of evidence for each main outcome; consider their relevance to key groups (e.g., healthcare providers, users, and policy makers).	19
Limitations	25	Discuss limitations at study and outcome level (e.g., risk of bias), and at review-level (e.g., incomplete retrieval of identified research, reporting bias).	21+22
Conclusions	26	Provide a general interpretation of the results in the context of other evidence, and implications for future research.	22+23
**FUNDING**			
Funding	27	Describe sources of funding for the systematic review and other support (e.g., supply of data); role of funders for the systematic review.	23

Supplementary file 2 Search strategy

Database	Records identified	After de-duplication
embase.com	733	724
Medline Ovid	476	94
Web of science	457	187
Cochrane CENTRAL	261	53
Google scholar	200	121
Total	2127	1179

embase.com

(music/de OR 'music therapy'/de OR (music OR musical OR musicotherap*):ab,ti) AND ('sleep'/de OR 'night sleep'/de OR 'nonREM sleep'/de OR 'REM sleep'/de OR 'sleep induction'/de OR 'sleep pattern'/de OR 'sleep quality'/de OR 'sleep spindle'/de OR 'sleep stage'/de OR 'sleep time'/de OR 'sleep waking cycle'/de OR 'slow wave sleep'/de OR 'sleep disorder'/de OR 'insomnia'/de OR 'sleep parameters'/exp OR 'circadian rhythm'/de OR 'chronobiology'/de OR 'sleep hygiene'/de OR 'sleep deprivation'/de OR 'somnolence'/de OR 'sleep medicine'/exp OR (sleep* OR asleep* OR insomnia OR bedtime OR bed-time OR circadian* OR rem OR nonrem OR chronobiology* *OR somnolence**):ab,ti) NOT ([animals]/lim NOT [humans]/lim) NOT ([Conference Abstract]/lim) AND [English]/lim

Medline Ovid

(music/ OR "music therapy"/ OR (music OR musical OR musicotherap*).ab,ti.) AND (exp sleep/ OR Sleep Wake Disorders/ OR Sleep Deprivation/ OR Circadian Rhythm/ OR (sleep* OR asleep* OR insomnia OR bedtime OR bed-time OR circadian* OR rem OR nonrem OR chronobiology*).ab,ti.) NOT (exp animals/ NOT humans/) AND english.la.

Web of science

TS=(((music OR musical OR musicotherap*)) AND ((sleep* OR asleep* OR insomnia OR bedtime OR bed-time OR circadian* OR rem OR nonrem OR chronobiology*))) AND DT=(article) AND LA=(english)

Cochrane

((music OR musical OR musicotherap*):ab,ti) AND ((sleep* OR asleep* OR insomnia OR bedtime OR bed-time OR circadian* OR rem OR nonrem OR chronobiology*):ab,ti)

Google scholar

music|musical|musicotherapy sleep|asleep|insomnia|bedtime|"bed-time"|circadian|rem|nonrem|chronobiology

Supplementary file 3 Sleep questionnaire characteristics

	RCSQ[1]	VSH [2]	PSQI[3]
**Items**	5	15	24
**Item assessment method**	Visual analogue scale	Numeric rating scale	Multiple methods
**Aspects of sleep assessed**	- Sleep depth<br>- Sleep latency<br>- Number of awakening<br>- Efficiency<br>- Sleep quality	- Sleep disturbance (sleep latency, mid-sleep awakenings, soundness of sleep, and movement during sleep)<br>- Effectiveness (rest upon awakening, subjective quality of sleep, and total sleep period)	- Quality<br>- Latency<br>- Duration<br>- Efficiency<br>- Disturbance<br>- Medication<br>- Daytime sleep<br>- Dysfunction
**Timing**	Assess sleep of night before		
**Validated**	Yes	Yes	Yes
**Reliability coefficient**	0.90	0.82	0.70-0.83

PSQI = Pittsburgh sleep quality index; RCSQ = Richards-Campbell sleep questionnaire; VSH = Verran and Snyder-Halpern sleep scale

[1] Richards KC, et al. Measurement of sleep in critically ill patients. J Nurs Meas. 2000;8(2):131-44.
[2] Snyder-Halpern R, Verran JA. Instrumentation to describe subjective sleep characteristics in healthy subjects. Res Nurs Health. 1987;10(3):155-63.
[3] Mollayeva T, et al. The Pittsburgh sleep quality index as a screening tool for sleep dysfunction in clinical and non-clinical samples: A systematic review and meta-analysis. Sleep Med Rev. 2016;25:52-73.
