# Supplementary material for: Music intervention for sleep quality in critically ill and surgical patients: a meta-analysis
Source: BMJ Open. 2021 May 10;11(5):e042510. doi: 10.1136/bmjopen-2020-042510 (PMC8112429; doi:10.1136/bmjopen-2020-042510)
Supplement: Author's manuscript [file bmjopen-2020-042510.draft_revisions.pdf]

BMJ Open

BMJ Open is committed to open peer review. As part of this commitment we make the peer review history of every article we publish publicly available.

When an article is published we post the peer reviewers' comments and the authors' responses online. We also post the versions of the paper that were used during peer review. These are the versions that the peer review comments apply to.

The versions of the paper that follow are the versions that were submitted during the peer review process. They are not the versions of record or the final published versions. They should not be cited or distributed as the published version of this manuscript.

BMJ Open is an open access journal and the full, final, typeset and author-corrected version of record of the manuscript is available on our site with no access controls, subscription charges or pay-per-view fees (http://bmjopen.bmj.com).

If you have any questions on BMJ Open's open peer review process please email info.bmjopen@bmj.com

BMJ Open

Music intervention for sleep quality in critically ill and surgical patients: a meta-analysis

Journal:	*BMJ Open*
Manuscript ID	bmjopen-2020-042510
Article Type:	Original research
Date Submitted by the Author:	07-Jul-2020
Complete List of Authors:	Kakar, Ellaha; Erasmus Medical Center, Neuroscience
Keywords:	INTENSIVE & CRITICAL CARE, SURGERY, SLEEP MEDICINE

SCHOLARONE™
Manuscripts

BMJ

I, the Submitting Author has the right to grant and does grant on behalf of all authors of the Work (as defined in the below author licence), an exclusive licence and/or a non-exclusive licence for contributions from authors who are: i) UK Crown employees; ii) where BMJ has agreed a CC-BY licence shall apply, and/or iii) in accordance with the terms applicable for US Federal Government officers or employees acting as part of their official duties; on a worldwide, perpetual, irrevocable, royalty-free basis to BMJ Publishing Group Ltd ("BMJ") its licensees and where the relevant Journal is co-owned by BMJ to the co-owners of the Journal, to publish the Work in this journal and any other BMJ products and to exploit all rights, as set out in our licence.

The Submitting Author accepts and understands that any supply made under these terms is made by BMJ to the Submitting Author unless you are acting as an employee on behalf of your employer or a postgraduate student of an affiliated institution which is paying any applicable article publishing charge ("APC") for Open Access articles. Where the Submitting Author wishes to make the Work available on an Open Access basis (and intends to pay the relevant APC), the terms of reuse of such Open Access shall be governed by a Creative Commons licence – details of these licences and which Creative Commons licence will apply to this Work are set out in our licence referred to above.

Other than as permitted in any relevant BMJ Author's Self Archiving Policies, I confirm this Work has not been accepted for publication elsewhere, is not being considered for publication elsewhere and does not duplicate material already published. I confirm all authors consent to publication of this Work and authorise the granting of this licence.

For peer review only

Music intervention for sleep quality in critically ill and surgical patients: a meta-analysis

E. Kakar, MD,[1,2*✉] , E. Venema, BSc,[3*] M. van der Jagt, MD, PhD,[4] M. Klimek, MD, PhD, [5] J. Jeekel, MD, PhD[1,2]

* shared first author

✉ Corresponding Author; Miss Ellaha Kakar, address: Doctor Molewaterplein 40, 3015 GD Rotterdam Room NA-2123, phone: +31(0)10 704 35 41, email: e.kakar@erasmusmc.nl

[1] Department of Surgery, Erasmus MC, University Medical Center Rotterdam, the Netherlands

[2] Department of Neuroscience, Erasmus MC, University Medical Center Rotterdam, the Netherlands

[3] Maastricht University, Maastricht, the Netherlands

[4] Department of Intensive Care Unit, Erasmus MC, University Medical Center Rotterdam, the Netherlands

[5] Department of Anesthesiology, Erasmus MC, University Medical Center Rotterdam, the Netherlands

For peer review only - http://bmjopen.bmj.com/site/about/guidelines.xhtml

Abstract

Objective Sleep disruption occurs frequently in hospitalized patients. Given the potential of music intervention as a non-pharmacological measure to improve sleep quality, we aimed to assess and quantify current literature on the effect of perioperative recorded music interventions on sleep quality and quantity in the adult critical care and surgical populations.

Design Systematic review and meta-analysis.

Data sources Embase, Medline Ovid, Cochrane Central, Web of Science, and Google Scholar.

Eligibility criteria for studies Randomized controlled trials assessing the effect of music on sleep quality in critically ill and surgical patients.

Methods The electronic databases were systematically searched. Data were screened, extracted, and appraised by two independent reviewers. Primary outcomes were sleep quality and quantity. The PRISMA guidelines were followed. Random effects meta-analysis was performed, and pooled standardized mean differences (SMD) with 95% confidence intervals (CI) were reported.

Results Five studies were included in qualitative and quantitative analysis (259 patients). Pooled data showed a significant overall effect of recorded music on sleep quality in the critical care and surgical population (SMD= 1.21 [95% CI 0.50; 1.91], $P < 0.01$), corresponding to a 26.0% [95% CI 19.0; 40.3] increase in sleep quality. A significant increase in sleep quantity of 36 minutes was found in one study.

Conclusions Recorded music showed a significant improvement in sleep quality in some critical care and surgical populations. Therefore, its use may be relevant to improve sleep, but given the moderate potential for bias in these studies, more studies are needed.

PROSPERO registration number CRD42020167783

Keywords Sleep, Music, Critical Care, Surgery

For peer review only

Article Summary

Strengths and limitations of this study

- This is the first comprehensive systematic review that studies the effect of music on sleep quality in critically ill and surgical patients.
- Patient reported outcomes, which are of increasing interest and of clinical relevance, are assessed in qualitative and quantitative analyses.
- Only data from randomized controlled trials are included.
- The risk of bias is moderate to high and the heterogeneity is high, which makes it difficult to draw definite conclusions.

For peer review only

Introduction

Sleep disruption is common in hospitalized patients.[1-4] A large cross-sectional study in the Netherlands found that sleep quality and quantity were significantly affected in hospitalized patients compared with patients' habitual sleep at home.[5] Sleep disruption is prevalent in almost all surgical and critically ill patients.[5-8] Important preventable factors associated with sleep disruption are pain, noise, anxiety, stress, sedative and analgesic medication usage, immobility, and severity of illness.[1, 5, 9-12]

In critically ill and surgical patients poor sleep has been associated with postoperative delirium, cardiovascular events in high-risk patients, post-operative fatigue (leading to prolonged immobility), altered mental status, [13-16] prolonged mechanical ventilation, cognitive impairment, altered immune function, and long term psychological comorbidities.[17] Thus, improving sleep promotes good health by improving emotional (anxiety, stress)[18] and possibly physical recovery. This is especially important for intensive care unit (ICU) and surgical patients given the loss of tissue integrity.[19, 20] Interventions in the hospital to improve sleep quality mainly include pharmacological interventions, such as benzodiazepines.[21-25] The Clinical Practice Guidelines for the Prevention and Management of Pain, Agitation/Sedation, Delirium, Immobility, and Sleep Disruption (PADIS) in Adult Patients in the ICU strongly recommend avoiding these drugs, given their potential to induce delirium and substance dependence.[17, 26, 27] Therefore, non-pharmacological measures are advised to prevent sleep disruption in hospitalized patients.[17, 28, 29 30-36]

Recently, several studies have shown a significant positive effect of perioperative music interventions on anxiety, pain, sedative and analgesic medication requirement, and neurohormonal stress response.[37-39] Also, in the ICU population associations have been made between music interventions and decreased serum cortisol levels, positive effects on state

For peer review only - http://bmjopen.bmj.com/site/about/guidelines.xhtml

anxiety, reduction in respiratory rate and systolic blood pressure, and decrease in sedative and analgesic requirement in mechanically ventilated patients.[40, 41]

Given the potential of music intervention as a non-pharmacological measure to improve sleep quality, we aimed to assess and quantify current literature on the effect of perioperative recorded music interventions on sleep quality and quantity in the adult critical care and surgical populations.

For peer review only

For peer review only - http://bmjopen.bmj.com/site/about/guidelines.xhtml

Materials and methods

Protocol and registration

This systematic review and meta-analysis follows the Preferred Reporting Items for Systematic Reviews and Meta-analysis (PRISMA) guidelines (supplementary file 1) and was registered in the PROSPERO database (https://www.crd.york.ac.uk/PROSPERO, as record number CRD42020167783).[42]

Search strategy

A systematic search was performed together with a dedicated biomedical information specialist in the Embase, Medline Ovid, Cochrane Central, Web of Science, and Google Scholar databases using a standardized protocol[43], including articles between the 1st of January 1981 (the year in which the first minimally invasive surgical intervention was performed, in order to avoid old literature which is not compatible with the current standard practice) and 27th of January 2020. The full search strategy is given in supplementary file 2. Keywords included "music" and "sleep".

Study screening and selection

Two reviewers (EK/EV) independently screened results of the search strategy on title/abstract, to confirm adherence to the eligibility criteria. Studies were eligible when they investigated (P) critically ill and/or surgical patients aged 18 years or older receiving a (I) recorded music intervention (C) compared to a control group in order to assess the effect on (O) sleep quality and quantity in (S) a randomized clinical trial (RCT). Critically ill populations included patients admitted to ICU or cardiac care unit (CCU), since CCU patients were also considered to have (potential) vital organ failure. Other eligibility criteria were; full text available and the study included in-hospital patients. Studies involving live music were excluded. Music intervention was defined as the use of recorded music consisting of melody, harmony, and rhythm. Nature

For peer review only - http://bmjopen.bmj.com/site/about/guidelines.xhtml

sounds were only included when used in addition to music. If studies compared music to multiple groups, the group without music most similar to the music group was chosen as control group (e.g. if groups were 'scheduled rest' and 'standard care', 'scheduled rest' was chosen as control group if the music group also received a resting period). Full text articles were discussed for admissibility. All disagreements between reviewers were resolved by discussion.

Data collection process and items

Data were extracted and checked by the same two reviewers (EK/EV) independently according to a pre-designed dataset. The following study characteristics were extracted: author, year of publication, study type, country of study, reason for admission (e.g. surgery type), population size, age (mean and SD), gender (% male), type of sleep assessment, timing of the intervention, setting (e.g. surgical ward, ICU, CCU), method of intervention delivery (e.g. headphones, CD-player), frequency and duration of the intervention, total duration of the intervention, music choice (e.g. patient- or researcher-selected), type of control group, and number of participants in the intervention and control group. Primary outcome measures were sleep quality and quantity measured after intervention or at the end of study period for both the control and intervention group. If a study applied music intervention and collected data at multiple time points only the final time point was used, since music could have a cumulative effect.

Risk of bias assessment

Risk of bias within studies was assessed independently by two reviewers (EK/EV) using the Cochrane Risk of Bias tool for RCTs.[44] Incomplete data of ≥ 10% due to drop-out (attrition bias) was considered as high risk. If the study protocol was not available, the risk for selective reporting was considered as unclear. Publication bias was assessed by creating funnel plots and the risk of bias across studies of the effect of music on sleep quality was assessed using

For peer review only - http://bmjopen.bmj.com/site/about/guidelines.xhtml

the GRADE (Grading of Recommendations, Assessment, Development and Evaluations) criteria.[44, 45]

Statistical analysis

Descriptive statistics were presented as means and their standard deviations (SD), counts (N), and percentages (%). For categorical age groups (e.g. age 20-50 = 8 patients, 51-60 = 7 patients) an approximation of the mean was calculated by calculating the mean for each category and pooling these means. Age ranges of the samples were used to calculate approximations for the SD by calculation one-fourth of the range of the data ((maximum-minimum)/4).[46] Studies were included for quantitative analysis if mean values and SDs of the sleep scores were reported. The primary analysis included meta-analysis for sleep quality and quantity. Standardized mean differences (SMD) were calculated as summary statistics of the main outcome and a random effects model was used to calculate the overall effect of music on sleep quality and quantity, accounting for between-study heterogeneity. The in between study-variance was estimated using the restricted maximum likelihood (REML) method. The level of heterogeneity was assessed using the I^2 statistic. For clinical interpretation the effect of the music intervention on sleep quality scores was expressed as a relative percentage increase, using a back-transformation of the acquired SMD that was described by the Cochrane Handbook for Systematic Reviews.[44] For this calculation, the SDs of the control groups were pooled to approximate the amount of variation in our patient population. Additionally the SD of a study with a large sample assessing sleep was used to make a more accurate approximation of the amount of variation. Data were analyzed using R version 3.6.3, and a two-sided p- value of < 0.05 was considered statistically significant.

For peer review only - http://bmjopen.bmj.com/site/about/guidelines.xhtml

Results

The search yielded 2127 articles leaving 1179 articles after removal of duplicates, and 1146 articles after removal of articles before the year 1981. Titles and abstracts screening resulted in leaving ten articles for full text assessment. Five studies were excluded for the following reasons; full text not available, use of guided imaging in combination with music, use of nature sounds, and use of multiple interventions. Subsequently, a total of five RCTs[47-51] were included in the qualitative and quantitative analysis (259 patients) (Figure 1).

Study characteristics

A detailed overview of patient and music intervention characteristics is presented in Table 1 and 2. Two studies were conducted at the ICU, two studies were conducted at the CCU and one study was performed in the surgical ward directly after ICU discharge. Apart from this study (Zimmerman et al.[51]) no other surgical papers were found. The mean age of the study population was 62.4 years with a predominance of males (65.8 %). Hansen et al.[47] and Su et al.[50] conducted the study at the ICU. The study of Su et al.[50] included medical ICU patients and the study of Hansen et al.[47] surgical (59%) and medical (41%) ICU patients. Cheraghi et al.[48] and Ryu et al.[49] conducted their study at the CCU including ACS and percutaneous transluminal coronary angiography (PTCA) patients. The study of Zimmerman et al.[51] was performed in the nursing ward after coronary artery bypass graft (CABG) surgery. The paper of Cheraghi et al.[48] was only available in the Persian language, the data extraction was assessed by a statistician in the Erasmus MC with Persian as mother language.

Sleep quality was measured using the Richards-Campbell Sleep Questionnaire (RCSQ, 40%), the Verran and Snyder-Halpern sleep scale (VSH, 40%), and the Pittsburgh Sleep Quality Index (PSQI, 20%). Only in the study of Ryu et al. sleep quantity was measured using patient questionnaires.

For peer review only - http://bmjopen.bmj.com/site/about/guidelines.xhtml

Music was mostly described as being soothing, sleep inducing, or sedating (60%).[47, 49, 50] Music was selected by the researcher in four of the five studies (80%), only Zimmerman et al. provided the option of choice between five audiotapes according to the patients' preference (Table 2).[51] Music was mostly administered using head- or earphones (60%). Duration of the intervention ranged from 30 to 53 minutes per day. Cheraghi et al.[48] administered the music intervention during three consecutive evenings, just before bedtime, after admission to the CCU. In the study of Su et al.[50] the intervention was only administered in the night of day three after admission to the ICU. Both studies assessed sleep quality in the morning of the consecutive day (day four). Hansen et al. applied the intervention during the day on day three after admission and assessed sleep immediately afterwards. Zimmerman et al.[51] applied the intervention on the night of POD two and assessed sleep the next morning (POD three). Ryu et al.[49] did not specify on which POD the music intervention and sleep assessment was performed. Control groups received either standard care (2 studies), scheduled rest (2 studies), or standard care with earplugs and eye shields (1 study).

Effect of music on sleep quality

Sleep quality scores of the final music session were pooled.[47-51] For the data of Cheraghi et al.[48] we applied reverse scoring since they used the PSQI, which was the only tool assessing sleep quality as better when scored lower on the scale. Pooling data resulted in an overall significant effect of recorded music on sleep quality in critical care and surgical patients (SMD= 1.21 [95% CI 0.50; 1.91], $p < 0.01$). Figure 2 presents the forest and funnel plot. The study of Cheraghi et al. showed a relative large effect compared to the other studies. Pooled data without the study of Cheraghi et al. the effect remained significant (SMD = 0.87 [95% CI 0.45; 1.29], $p < 0.01$).

For the clinical interpretation we performed a back-transformation based on a recent Dutch study which assessed sleep in 194 patient using the RCSQ, which is a validated and reliable

For peer review only - http://bmjopen.bmj.com/site/about/guidelines.xhtml

sleep quality assessment tool in the critically ill.[52] The reported median inter quartile range (IQR) for the RCSQ reported in this study was transformed to mean ± SD using the methodology of Wan et al.[46] This resulted in an SD approximation of 2.24. Using the effect size of our meta-analysis, SMD = 1.21, this resulted in a reduction of 2.71 [95% CI 1.12; 4.28] on the RCSQ which ranges from zero to ten. This implies a 27.1% [95% CI 11.2; 42.8] increase in sleep quality in the ICU due to a music intervention. In order to check if the effect in our study population approximates the effect calculated above we also applied the back-transformation using the pooled RCSQ SDs (N = 51, SD = 2.53) of the control groups in our study. This resulted in a reduction of 3.06 [95% CI 1.27; 4.83] on the RCSQ, equal to 30.6% [95% CI 12.7; 48.3] increase in sleep quality.

Effect of music on sleep quantity

Meta-analysis on the effect of music on sleep quantity could not be performed since it was only assessed by the study of Ryu et al in PTCA patients.[49] This study found a statistically significant higher sleep duration in the music group (N = 29) compared to the control group (N = 29); 279.31 ± 43.99 respectively 243.10 ± 42.68 with a p- value of 0.002.

Risk of bias assessment

The overall risk of bias was moderate to high (Figure 3). All included studies reported the use of randomization, but a high risk of selection bias was considered due to insufficient details regarding random sequence generation in two studies[47, 51], and allocation concealment in four studies.[48-51] Due to the type of intervention and the subjective outcome assessment blinding of participants and personnel was not possible, therefore performance and detection bias were considered high in all studies. Attrition bias was considered low in all studies since none of the studies had a drop-out rate of ≥10%. A full study protocol was missing (assessed by checking reported registration number of the trial in the paper and registers for clinical trials (e.g.

For peer review only - http://bmjopen.bmj.com/site/about/guidelines.xhtml

clinicaltrials.gov) for all included studies leading to an unclear risk for reporting bias. Two included studies (20%) had a high risk of bias due to other reasons; Cheraghi et al.[48] excluded patients if listening to the music induced anxiety or brought up bad memories, which could have contributed to an overestimation of the effect of music intervention on sleep quality, and Hansen et al.[47] measured sleep quality during daytime rest with the RCSQ which is only a validated measurement tool for sleep quality after nocturnal sleep. Figure 4 presents the summary of the risk of bias assessment.

Grade certainty rating

Risk of bias was moderate to high for sleep quality. Imprecision was considered medium since the effect size in the 95% CI of the pooled estimate ranged from medium to high, which could influence clinical decision-making. Consistency was considered moderate to high since individual studies included in the meta-analysis showed a positive influence of music on sleep quality, the 95% CIs overlapped, but the heterogeneity was high. Heterogeneity was high and statistically significant for sleep quality (I^2 = 85% [95% CI 68%; 93%, p < 0.01), possibly caused by the differences in patient characteristics. Meta-regression, to assess the reason for high heterogeneity, could not be performed since there was a limited number of papers available. Directness was considered high as studies directly investigated music in the population of interest, the intervention was mostly applied on the third day after ICU/CCU admission or after surgery, and the reported outcomes were critical for decision-making (e.g. if sleep quality was low, administration of sleep medication could be considered). Scattering in the funnel plot is symmetrical, therefore the risk for publication bias is considered low. In conclusion, we rate the GRADE certainty rating as moderate.

For peer review only - http://bmjopen.bmj.com/site/about/guidelines.xhtml

Discussion

This meta-analysis showed a significant beneficial effect of recorded music intervention on sleep quality in critically ill and post-CABG patients admitted to the ward. These findings are in line with the current literature.[29, 53-55] Hu et al. reviewed literature for the effect of non-pharmacological interventions on sleep quality in the ICU.[29] Although, they could not pool data and their quality of evidence was low, they found that music intervention might positively influence sleep quality. Also, Feng et al. studied the effect of music and other non-pharmacological interventions (e.g. music combined with other interventions, acupuncture) on primary insomnia (insomnia after ruling out several other conditions such as psychiatric (depression and anxiety), medical (pain), circadian (phase-delay syndrome), or other sleep disorders. They found that solely music intervention had the highest ranking and seemed to offer clear advantages on sleep quality.[53]

Sleep quality has been an important focus in the current society since it is an essential part of good health.[18, 20] Health care professionals should be aware of the detrimental consequences of sleep disturbances. As mentioned before, poor sleep quality is associated with several important risks. [56-59] Sleep architecture in critical care patients is characterized by decreased total sleep time (TST), sleep divided into short discrete episodes, sleep occurring mostly during daytime, increased amount of light sleep stages and decreased amount of deeper sleep stage.[8, 17, 60-63] This inevitably leads to loss of the restorative and resting function of sleep, which is achieved by deeper sleep stages.[40, 60, 62, 64] Sleep disturbances have been associated with cognitive impairment, including delirium, prolonged duration of mechanical ventilation, altered immune function, and long-term psychological comorbidities.[8, 17, 65-68] Furthermore, acutely developed sleep disturbances in the ICU can persist for a longer time frame after discharge and is the most common mentioned stressful factor experienced by patients.[60, 69-72] Nevertheless, the administration of sedative and analgesic medication, used to improve patient comfort, may

For peer review only - http://bmjopen.bmj.com/site/about/guidelines.xhtml

negatively impact sleep quality.[5, 10-12, 73-77] Considering the above, preventing sleep disturbances in critical care patients is of great importance for patient related outcomes.

We made an approximation of 27.1% increase in sleep quality if music intervention is applied based on our pooled data. Several studies comparing benzodiazepines to a placebo showed even lower effects, ranging from 12.9% to 21.4% improvement of sleep quality. [78] [79] [80] [81] This indicates that music may have a similar or even better effect on sleep quality compared with drug therapy with benzodiazepines, while the patient is not exposed to their harmful side-effects and risk of dependency. Therefore, the use of recorded music in hospitalized patients can be considered a clinically relevant intervention. Only one paper in this study assessed effect of music on sleep quantity and found a significant increase in sleep duration of 36 min. in a sample of 58 patients.[49] Krenk et al. studied the effect of Zolpidem on TST and found it was 28 min. shorter compared to the placebo group, this finding was not significant.[80] Simons et al. found a significant extension of TST with 57 min. when using Temazepam in healthy volunteers.[81] Also, Sharma et al. measured an increase of TST when Zolpidem was used, unfortunately both studies did not assess or find effect on deeper sleep stages. Extension of the TST does not necessarily lead to optimal sleep, since, as mentioned before, extension of the deeper sleep stages (restorative and resting function) are of more importance. Therefore, it is difficult to draw conclusions regarding sleep duration in this study.

Until now the physiology behind the effect of music on sleep remains unclear. Several theories have been proposed on the effect of music on sleep including; rhythmic entrainment, masking, and distraction.[55] It is important to recognize that the effect of music is complex and also plays an important role on neuro- and psychophysiological levels.[82, 83] Literature indicates that music induces the anxiolytic effect through suppression of the sympathetic nervous system. This leads to suppression of the adrenergic activity and thus reduction of the stress hormone adrenaline.[84,]

For peer review only - http://bmjopen.bmj.com/site/about/guidelines.xhtml

For peer review only

[85] Also, music stimulates the release of endorphins by activating memory and the limbic system, which plays an important role in the sense of emotional well-being.[86, 87] More specifically, the nucleus accumbens is activated leading to increased dopamine release and deactivation of areas in the brain related to stress and cortisol signaling.[88] Furthermore, several studies have suggested positive effects of vagus nerve stimulation (VNS) [89] on objective and subjective sleep parameters in epilepsy patients,[90, 91] which may also be induced by music via the auricular branch of the vagal nerve. VNS positively influences sleep architecture by increasing sleep latency (indicating positive effect on daytime sleepiness) and slow wave sleep (indicating increased amount of deeper sleep stages). Erk et al. (2010) and Pandya et al. (2012) proposed that the effect of VNS might work via the afferents of the vagal nerve through the nucleus tractus solitarius in the brain. The nucleus tractus solitatius has then connections with the amygdala, frontal cortex, and other regions in the brain that influence cognitive emotion regulation.[92, 93] These theories are the basis on which music interventions might work. Also, sleep disturbances are multifactorial, and thus non-pharmacological interventions should focus on tackling multiple factors affecting sleep at the same time. Since music intervention has already been proven effective on several factors causing sleep disturbances (anxiety, pain, stress etc.), the beneficial effects found in this study seem valid.

The GRADE certainty was rated moderate, which implies that the authors believe the estimated clinical relevant effects are probably close to the true effect. Also, the effect lies probably near the true effect since the effect found is large and there is at least a moderate effect of music on sleep quality based on the lower limit of the 95% CI of the effect estimate (0.50). Unfortunately, the relatively small number of studies and sample sizes lead to uncertain reliability and validity of this study, which is also seen in the current literature regarding the effect of music on sleep in other populations.[78-80]

For peer review only - http://bmjopen.bmj.com/site/about/guidelines.xhtml

Based on the previous arguments, we suggest future research should consist of high quality randomized clinical trials with the use of objective tools for sleep assessment, as recorded music seems effective and clinically relevant, in order to make more definite conclusions regarding the effect of recorded music on sleep quality and quantity in hospitalized critical care and surgical patients.

Strengths and limitations

The sleep assessment tools used in the included studies of this meta-analysis were all validated, reliable, and easily applicable tools for sleep quality assessment. An important limitation of this review is the limited amount of studies included for quantitative analysis, while it is unlikely we missed studies meeting our inclusion criteria since the search was extensive and was performed by a biomedical information specialist. Second, heterogeneity was high. Third, there was a moderate to high risk of bias, due to insufficient information on random sequence generation, allocation concealment, and that the primary outcome included subjective patient reported questionnaires and blinding of participants and personnel in a music intervention study is not feasible. Chlan et al. (2013) demonstrated in a large clinical trial with 373 patients, randomized to three groups (music intervention, noise-cancelling headphones, and usual care), a consistent decrease in anxiety in the music group compared to noise-cancelling headphones and usual care groups.[94] In this trial, bias due to non-blinding of the outcome assessors was minimized by including an extra control group wearing noise-cancelling headphones (without music). In line with this study, it seems less likely that the effects of music are overestimated compared with the control group without headphones and music intervention.

For peer review only - http://bmjopen.bmj.com/site/about/guidelines.xhtml

Conclusion

This systematic review and meta-analysis of randomized controlled trials showed that recorded music interventions significantly increase sleep quality in the critically ill, ACS or PTCA patients and after cardiac surgery for coronary artery disease. Music is easily applicable and has no risks and side-effects and should therefore be considered as a suitable non-medicinal alternative for sleep quality improvement in these patient groups. Since the clinical trials performed until now are small and of low quality, we suggest larger and high quality randomized clinical trials for future research, including broader patient populations.

Acknowledgements

The authors thank W. Bramer, biomedical information specialist of the Medical Library at the Erasmus University Medical Centre, for his assistance in the literature search and J. van Rosmalen, assistant professor at the Department of Biostatistics of the Erasmus University Medical Centre, for his assistance in the statistical analysis.

Funding This research received no specific grant from any funding agency in the public, commercial or not-for-profit sectors.

Disclosure The authors declare no other conflict of interest.

JJ conceived the study idea. EK and EV coordinated the systematic review. EK and EV screened abstracts and full texts. EK and EV wrote the first draft of the manuscript and judged risk of bias in the studies. EK, EV, MJ, MK, and JJ interpreted the data. EK, EV, MJ, MK, and JJ critically revised the manuscript. EK, EV, MJ, MK, and JJ had full access to all of the data in the study and can take responsibility for the integrity of the data and the accuracy of the data analysis.

References

For peer review only

For peer review only - http://bmjopen.bmj.com/site/about/guidelines.xhtml

1. Dobing S, et al. Sleep Quality and Factors Influencing Self-Reported Sleep Duration and Quality in the General Internal Medicine Inpatient Population. PLoS One. 2016;11(6):e0156735.
2. Dogan O, et al. Sleep quality in hospitalized patients. J Clin Nurs. 2005;14(1):107-13.
3. Jakobsen G, et al. Sleep quality in hospitalized patients with advanced cancer: an observational study using self-reports of sleep and actigraphy. Support Care Cancer. 2020;28(4):2015-23.
4. Park MJ, Kim KH. What affects the subjective sleep quality of hospitalized elderly patients? Geriatr Gerontol Int. 2017;17(3):471-9.
5. Wesselius HM, et al. Quality and Quantity of Sleep and Factors Associated With Sleep Disturbance in Hospitalized Patients. JAMA Intern Med. 2018;178(9):1201-8.
6. Cilingir D, et al. Factors affecting the sleep status of surgical and medical patients at a University Hospital of Turkey. J Pak Med Assoc. 2016;66(12):1535-40.
7. Bani Younis M, Hayajneh FA. Quality of Sleep Among Intensive Care Unit Patients: A Literature Review. Crit Care Nurs Q. 2018;41(2):170-7.
8. Pulak LM, Jensen L. Sleep in the Intensive Care Unit: A Review. J Intensive Care Med. 2016;31(1):14-23.
9. Parthasarathy S, et al. Biological validity to sleep measurements during critical illness. Crit Care Med. 2010;38(2):705-6.
10. DeMartinis NA, et al. New approaches for the treatment of sleep disorders. Adv Pharmacol. 2009;57:187-235.
11. Yilmaz M, et al. Sleep quality of hospitalized patients in surgical units. Nurs Forum. 2012;47(3):183-92.
12. Klemann N, et al. Factors affecting post-operative sleep in patients undergoing colorectal surgery - a systematic review. Dan Med J. 2015;62(4):A5053.
13. Su X, Wang DX. Improve postoperative sleep: what can we do? Curr Opin Anaesthesiol. 2018;31(1):83-8.
14. Kjolhede P, et al. The impact of quality of sleep on recovery from fast-track abdominal hysterectomy. J Clin Sleep Med. 2012;8(4):395-402.
15. Rosenberg J. Sleep disturbances after non-cardiac surgery. Sleep Med Rev. 2001;5(2):129-37.
16. Gogenur I. Postoperative circadian disturbances. Dan Med Bull. 2010;57(12):B4205.
17. Devlin JW, et al. Clinical Practice Guidelines for the Prevention and Management of Pain, Agitation/Sedation, Delirium, Immobility, and Sleep Disruption in Adult Patients in the ICU. Crit Care Med. 2018;46(9):e825-e73.
18. Lieberman HR, et al. Severe decrements in cognition function and mood induced by sleep loss, heat, dehydration, and undernutrition during simulated combat. Biol Psychiatry. 2005;57(4):422-9.
19. Telias I, Wilcox ME. Sleep and Circadian Rhythm in Critical Illness. Crit Care. 2019;23(1):82.
20. Peever J, Fuller PM. The Biology of REM Sleep. Curr Biol. 2017;27(22):R1237-R48.
21. Gillis CM, et al. Inpatient pharmacological sleep aid utilization is common at a tertiary medical center. J Hosp Med. 2014;9(10):652-7.
22. Frighetto L, et al. An assessment of quality of sleep and the use of drugs with sedating properties in hospitalized adult patients. Health Qual Life Outcomes. 2004;2:17.
23. van Beek S, et al. The effect of midazolam as premedication on the quality of postoperative recovery after laparotomy: a randomized clinical trial
Effet du midazolam en premedication sur la qualite de la recuperation postoperatoire apres une laparotomie : une etude clinique randomisee. Can J Anaesth. 2020;67(1):32-41.
24. Maurice-Szamburski A, et al. Effect of sedative premedication on patient experience after general anesthesia: a randomized clinical trial. JAMA. 2015;313(9):916-25.

For peer review only - http://bmjopen.bmj.com/site/about/guidelines.xhtml

25. Mijderwijk H, et al. Lorazepam does not improve the quality of recovery in day-case surgery patients: a randomised placebo-controlled clinical trial. Eur J Anaesthesiol. 2013;30(12):743-51.
26. Halfens R, et al. Effect of the use of sleep medication in Dutch hospitals on the use of sleep medication at home. J Adv Nurs. 1994;19(1):66-70.
27. Brummett CM, et al. New Persistent Opioid Use After Minor and Major Surgical Procedures in US Adults. JAMA Surg. 2017;152(6):e170504.
28. Lenhart SE, Buysse DJ. Treatment of insomnia in hospitalized patients. Ann Pharmacother. 2001;35(11):1449-57.
29. Hu RF, et al. Non-pharmacological interventions for sleep promotion in the intensive care unit. Cochrane Database Syst Rev. 2015(10):CD008808.
30. Brandt J, Leong C. Benzodiazepines and Z-Drugs: An Updated Review of Major Adverse Outcomes Reported on in Epidemiologic Research. Drugs R D. 2017;17(4):493-507.
31. Glass J, et al. Sedative hypnotics in older people with insomnia: meta-analysis of risks and benefits. BMJ. 2005;331(7526):1169.
32. Weinbroum AA, et al. The midazolam-induced paradox phenomenon is reversible by flumazenil. Epidemiology, patient characteristics and review of the literature. Eur J Anaesthesiol. 2001;18(12):789-97.
33. American Geriatrics Society Expert Panel on Postoperative Delirium in Older A. American Geriatrics Society abstracted clinical practice guideline for postoperative delirium in older adults. J Am Geriatr Soc. 2015;63(1):142-50.
34. Bringman H, et al. Relaxing music as pre-medication before surgery: a randomised controlled trial. Acta Anaesthesiol Scand. 2009;53(6):759-64.
35. Kassie GM, et al. Preoperative medication use and postoperative delirium: a systematic review. BMC Geriatr. 2017;17(1):298.
36. Zaal IJ, et al. Benzodiazepine-associated delirium in critically ill adults. Intensive Care Med. 2015;41(12):2130-7.
37. Fu VX, et al. The Effect of Perioperative Music on Medication Requirement and Hospital Length of Stay: A Meta-analysis. Ann Surg. 2019.
38. Fu VX, et al. The Effect of Perioperative Music on the Stress Response to Surgery: A Meta-analysis. J Surg Res. 2019;244:444-55.
39. Kuhlmann AYR, et al. Meta-analysis evaluating music interventions for anxiety and pain in surgery. Br J Surg. 2018;105(7):773-83.
40. Bradt J, Dileo C. Music interventions for mechanically ventilated patients. Cochrane Database Syst Rev. 2014(12):CD006902.
41. Khan SH, et al. Effects of music intervention on inflammatory markers in critically ill and post-operative patients: A systematic review of the literature. Heart Lung. 2018;47(5):489-96.
42. Moher D, et al. Preferred reporting items for systematic reviews and meta-analyses: the PRISMA statement. PLoS Med. 2009;6(7):e1000097.
43. Bramer WM, et al. Evaluation of a new method for librarian-mediated literature searches for systematic reviews. Res Synth Methods. 2018;9(4):510-20.
44. Higgins JPT GS. The Cochrane Handbook for Systematic Reviews of Interventions Version 5.1.0 2011.
45. Guyatt GH, et al. GRADE: an emerging consensus on rating quality of evidence and strength of recommendations. BMJ. 2008;336(7650):924-6.
46. Wan X, et al. Estimating the sample mean and standard deviation from the sample size, median, range and/or interquartile range. BMC Med Res Methodol. 2014;14:135.
47. Hansen IP, et al. Effects of music during daytime rest in the intensive care unit. Nurs Crit Care. 2018;23(4):207-13.
48. Mali C, et al. The effect of instrumental music on sleep in patients admitted to Coronary Care Unit: sid.ir; 2015.

For peer review only

49. Ryu MJ, et al. Effect of sleep-inducing music on sleep in persons with percutaneous transluminal coronary angiography in the cardiac care unit. J Clin Nurs. 2012;21(5-6):728-35.
50. Su CP, et al. A randomized controlled trial of the effects of listening to non-commercial music on quality of nocturnal sleep and relaxation indices in patients in medical intensive care unit. J Adv Nurs. 2013;69(6):1377-89.
51. Zimmerman L, et al. The effects of music interventions on postoperative pain and sleep in coronary artery bypass graft (CABG) patients. Sch Inq Nurs Pract. 1996;10(2):153-70; discussion 71-4.
52. Rood P, et al. Development and daily use of a numeric rating score to assess sleep quality in ICU patients. J Crit Care. 2019;52:68-74.
53. Feng F, et al. Can music improve sleep quality in adults with primary insomnia? A systematic review and network meta-analysis. Int J Nurs Stud. 2018;77:189-96.
54. Bani Younis M, et al. Measurement and Nonpharmacologic Management of Sleep Disturbance in the Intensive Care Units: A Literature Review. Crit Care Nurs Q. 2019;42(1):75-80.
55. Dickson GT, Schubert E. How does music aid sleep? literature review. Sleep Med. 2019;63:142-50.
56. Hoevenaar-Blom MP, et al. Sleep duration and sleep quality in relation to 12-year cardiovascular disease incidence: the MORGEN study. Sleep. 2011;34(11):1487-92.
57. Gottlieb DJ, et al. Association of sleep time with diabetes mellitus and impaired glucose tolerance. Arch Intern Med. 2005;165(8):863-7.
58. Gamaldo CE, et al. The sleep-immunity relationship. Neurol Clin. 2012;30(4):1313-43.
59. Alvarez GG, Ayas NT. The impact of daily sleep duration on health: a review of the literature. Prog Cardiovasc Nurs. 2004;19(2):56-9.
60. Pisani MA, et al. Sleep in the intensive care unit. Am J Respir Crit Care Med. 2015;191(7):731-8.
61. Cooper AB, et al. Sleep in critically ill patients requiring mechanical ventilation. Chest. 2000;117(3):809-18.
62. Freedman NS, et al. Abnormal sleep/wake cycles and the effect of environmental noise on sleep disruption in the intensive care unit. Am J Respir Crit Care Med. 2001;163(2):451-7.
63. Elliott R, et al. Characterisation of sleep in intensive care using 24-hour polysomnography: an observational study. Crit Care. 2013;17(2):R46.
64. Boyko Y, et al. Sleep quality and circadian rhythm disruption in the intensive care unit: a review. Nat Sci Sleep. 2017;9:277-84.
65. Sanders RD, Maze M. Contribution of sedative-hypnotic agents to delirium via modulation of the sleep pathway. Can J Anaesth. 2011;58(2):149-56.
66. Friese RS. Sleep and recovery from critical illness and injury: a review of theory, current practice, and future directions. Crit Care Med. 2008;36(3):697-705.
67. Opp MR. Sleep and psychoneuroimmunology. Immunol Allergy Clin North Am. 2009;29(2):295-307.
68. Orwelius L, et al. Prevalence of sleep disturbances and long-term reduced health-related quality of life after critical care: a prospective multicenter cohort study. Crit Care. 2008;12(4):R97.
69. Tembo AC, et al. The experience of sleep deprivation in intensive care patients: findings from a larger hermeneutic phenomenological study. Intensive Crit Care Nurs. 2013;29(6):310-6.
70. Simini B. Patients' perceptions of intensive care. Lancet. 1999;354(9178):571-2.
71. Rotondi AJ, et al. Patients' recollections of stressful experiences while receiving prolonged mechanical ventilation in an intensive care unit. Crit Care Med. 2002;30(4):746-52.
72. Kamdar BB, et al. Sleep deprivation in critical illness: its role in physical and psychological recovery. J Intensive Care Med. 2012;27(2):97-111.

For peer review only

73. Barr J, et al. Clinical practice guidelines for the management of pain, agitation, and delirium in adult patients in the intensive care unit. Crit Care Med. 2013;41(1):263-306.
74. Hetland B, et al. The influence of music during mechanical ventilation and weaning from mechanical ventilation: A review. Heart Lung. 2015;44(5):416-25.
75. Jean R, et al. Effects of deep sedation on sleep in critically ill medical patients on mechanical ventilation. J Sleep Res. 2019:e12894.
76. Pandharipande P, Ely EW. Sedative and analgesic medications: risk factors for delirium and sleep disturbances in the critically ill. Crit Care Clin. 2006;22(2):313-27, vii.
77. Dolan R, et al. A prospective analysis of sleep deprivation and disturbance in surgical patients. Ann Med Surg (Lond). 2016;6:1-5.
78. Sharma MK, et al. Effects of zolpidem on sleep parameters in patients with cirrhosis and sleep disturbances: A randomized, placebo-controlled trial. Clin Mol Hepatol. 2019;25(2):199-209.
79. Huang Y, et al. The effect of zolpidem on sleep quality, stress status, and nondipping hypertension. Sleep Med. 2012;13(3):263-8.
80. Krenk L, et al. Postoperative sleep disturbances after zolpidem treatment in fast-track hip and knee replacement. J Clin Sleep Med. 2014;10(3):321-6.
81. Simons R, et al. Usefulness of temazepam and zaleplon to induce afternoon sleep. Mil Med. 2006;171(10):998-1001.
82. Koelsch S, et al. Adults and children processing music: an fMRI study. Neuroimage. 2005;25(4):1068-76.
83. Menon V, Levitin DJ. The rewards of music listening: response and physiological connectivity of the mesolimbic system. Neuroimage. 2005;28(1):175-84.
84. Chlan L. Effectiveness of a music therapy intervention on relaxation and anxiety for patients receiving ventilatory assistance. Heart Lung. 1998;27(3):169-76.
85. Gillen E, et al. Effects of music listening on adult patients' pre-procedural state anxiety in hospital. JBI Libr Syst Rev. 2008;6(17):686-732.
86. Beaulieu-Boire G, et al. Music and biological stress dampening in mechanically-ventilated patients at the intensive care unit ward-a prospective interventional randomized crossover trial. J Crit Care. 2013;28(4):442-50.
87. Tracy MF, Chlan L. Nonpharmacological interventions to manage common symptoms in patients receiving mechanical ventilation. Crit Care Nurse. 2011;31(3):19-28.
88. Chanda ML, Levitin DJ. The neurochemistry of music. Trends Cogn Sci. 2013;17(4):179-93.
89. Sigurdardottir GA, et al. A pilot study on high amplitude low frequency-music impulse stimulation as an add-on treatment for depression. Brain Behav. 2019;9(10):e01399.
90. Jain SV, Glauser TA. Effects of epilepsy treatments on sleep architecture and daytime sleepiness: an evidence-based review of objective sleep metrics. Epilepsia. 2014;55(1):26-37.
91. Romero-Osorio O, et al. Changes in sleep patterns after vagus nerve stimulation, deep brain stimulation or epilepsy surgery: Systematic review of the literature. Seizure. 2018;56:4-8.
92. Erk S, et al. Acute and sustained effects of cognitive emotion regulation in major depression. J Neurosci. 2010;30(47):15726-34.
93. Pandya M, et al. Where in the brain is depression? Curr Psychiatry Rep. 2012;14(6):634-42.
94. Chlan LL, et al. Effects of patient-directed music intervention on anxiety and sedative exposure in critically ill patients receiving mechanical ventilatory support: a randomized clinical trial. JAMA. 2013;309(22):2335-44.

For peer review only - http://bmjopen.bmj.com/site/about/guidelines.xhtml

Tables and figures

Figure 1. PRISMA Flow Diagram. N indicates the number of articles.

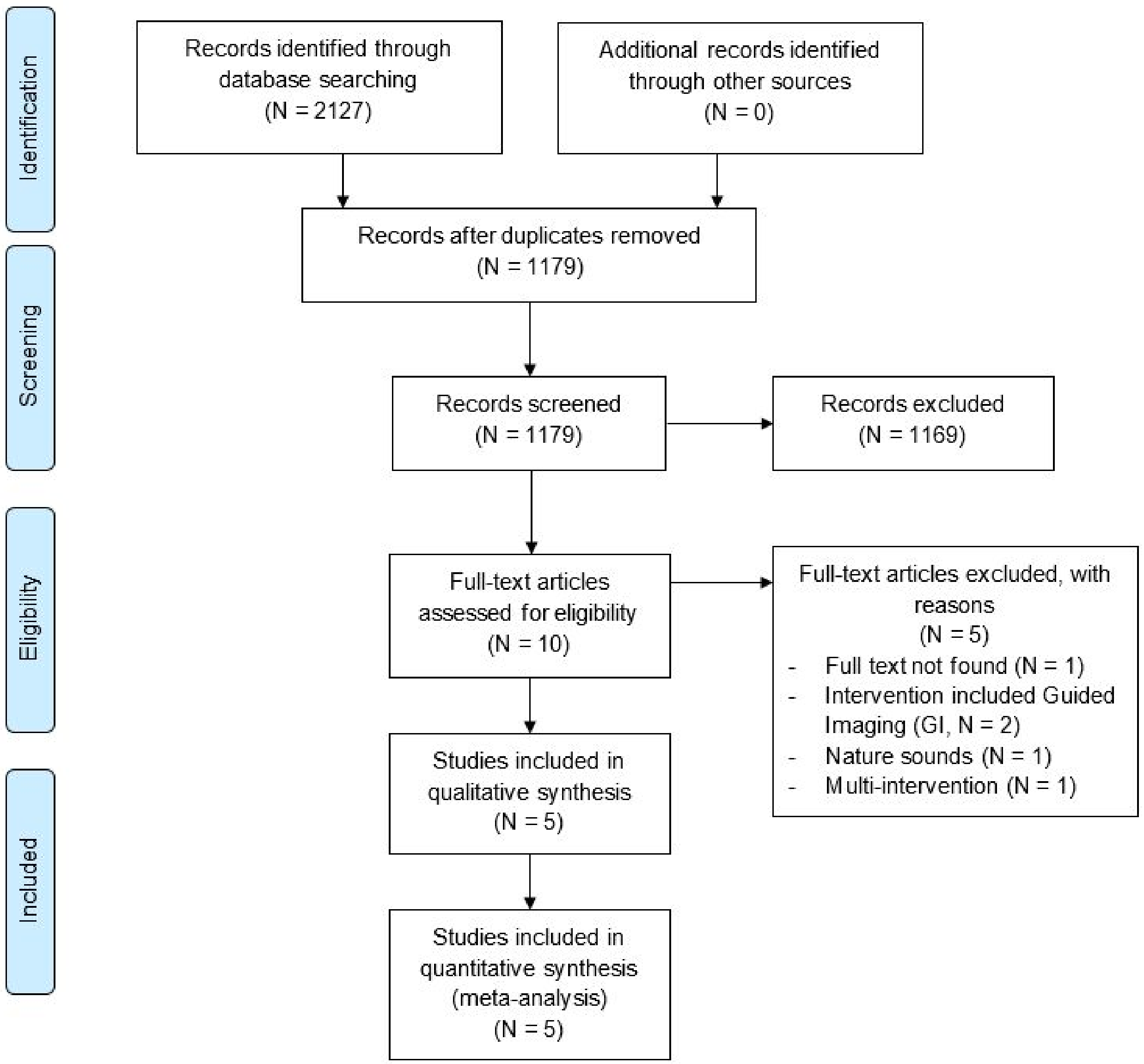

Table 1 Study characteristics

Study	Study type	Country	Reason for admission	Population size	Mean age (SD)		Gender (% male)		Outcome assessment
					Intervention	Control	Intervention	Control	
**Cheraghi, 2015**	RCT	Iran	ACS	72	56.9(10.5)	62.1(12.7)	80.6%	80.6%	Sleep quality (PSQI)
**Hansen, 2018**	RCT	Denmark	Surgical and medical ICU	37	60.0(18.0)	65(16.0)	55.6%	52.6%	Sleep quality (RCSQ)
**Ryu, 2011**	RCT	South Korea	PTCA	58	58.5(15.0)	59.9(15.0)	65.5%	65.5%	Sleep quality (VSH), sleep quantity (questionnaire)
**Su, 2012**	RCT	Taiwan	Medical ICU	28	62.4(9.1)	60.9(10.8)	57.1%	64.3%	Sleep quality (VSH and PSG)
**Zimmerman, 1996**	RCT	USA	CABG	64	67.0(9.9)	67.0(9.9)	68.0%	68.0%	Sleep quality (RCSQ)
**Summary of all studies**					60.8(12.6)	63.1(11.2)	68.0%	64.4%	

SD = standard deviation; RCT = randomized controlled trial; ICU = intensive care unit; ACS = acute coronary syndrome; PTCA = percutaneous transluminal coronary angioplasty; CABG = coronary artery bypass graft; PSQI = Pittsburgh sleep quality index; RCSQ = Richards-Campbell sleep questionnaire; VSH = Verran and Snyder-Halpern sleep scale; PSG = polysomnography; USA = United States of America

For peer review only

Table 2 Music intervention characteristics

Study	Timing intervention	Location	Medium	Frequency (per day) x duration (min.)	Total duration (min.)	Intervention	Interventio n choice	N intervention	Control	N control
**Cheraghi, 2015**	Every night after admission during 3 nights	CCU	Headphones	3 x 45	135	Non-vocal music, accepted by the society	Researcher	36	Standard care	36
**Hansen, 2018**	Day 3 after admission	ICU	Loudspeaker in the ceiling of patients' bed	1 x 30	30	MusiCure: soothing music, soft wind, bird twitter, ocean sound, music instruments	Researcher	18	Scheduled rest	19
**Ryu, 2011**	NR	CCU	Earphones	1 x 53	53	Sleep inducing music: nature sounds, delta wave control music, and Goldberg variations BWV + eye bandages	Researcher	29	Earplugs and eye-shield	29
**Su, 2012**	Day 3 after admission	ICU	CD player	1 x 45	45	Sedating piano music: smooth rhythm to achieve a relaxing effect, a tempo of 60-80 beats/min, minor tonalities, smooth melody lines and no dramatic changes in volume and rhythm	Researcher	14	Standard care	14
**Zimmerman, 1996**	POD 2-3	Ward	Headphones	2 x 30	60	Country western instrumental, fresh aire (Mannheim Steamroller), winter into spring (George Winston), prelude and comfort zone (Steven Halpern) (facilitates relaxation)	Option out of 5 audiotapes, in case of no preference Halpern was chosen	32	Scheduled rest	32

ICU = intensive care unit; CCU = coronary care unit; POD = postoperative day; NR = not reported

Figure 2. Forest and funnel plot assessing the effect of music on subjective sleep quality

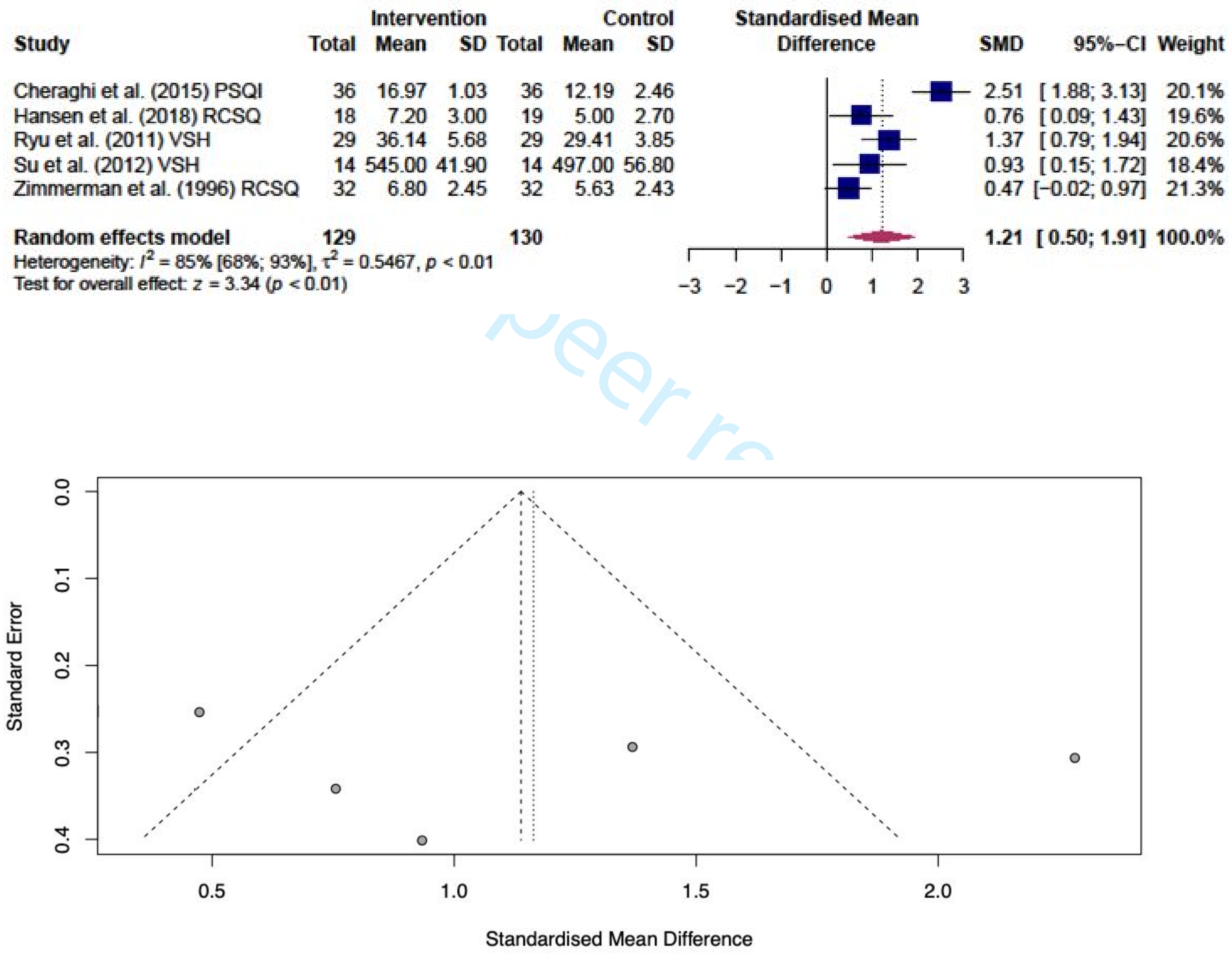

Figure 3. Risk of bias assessment

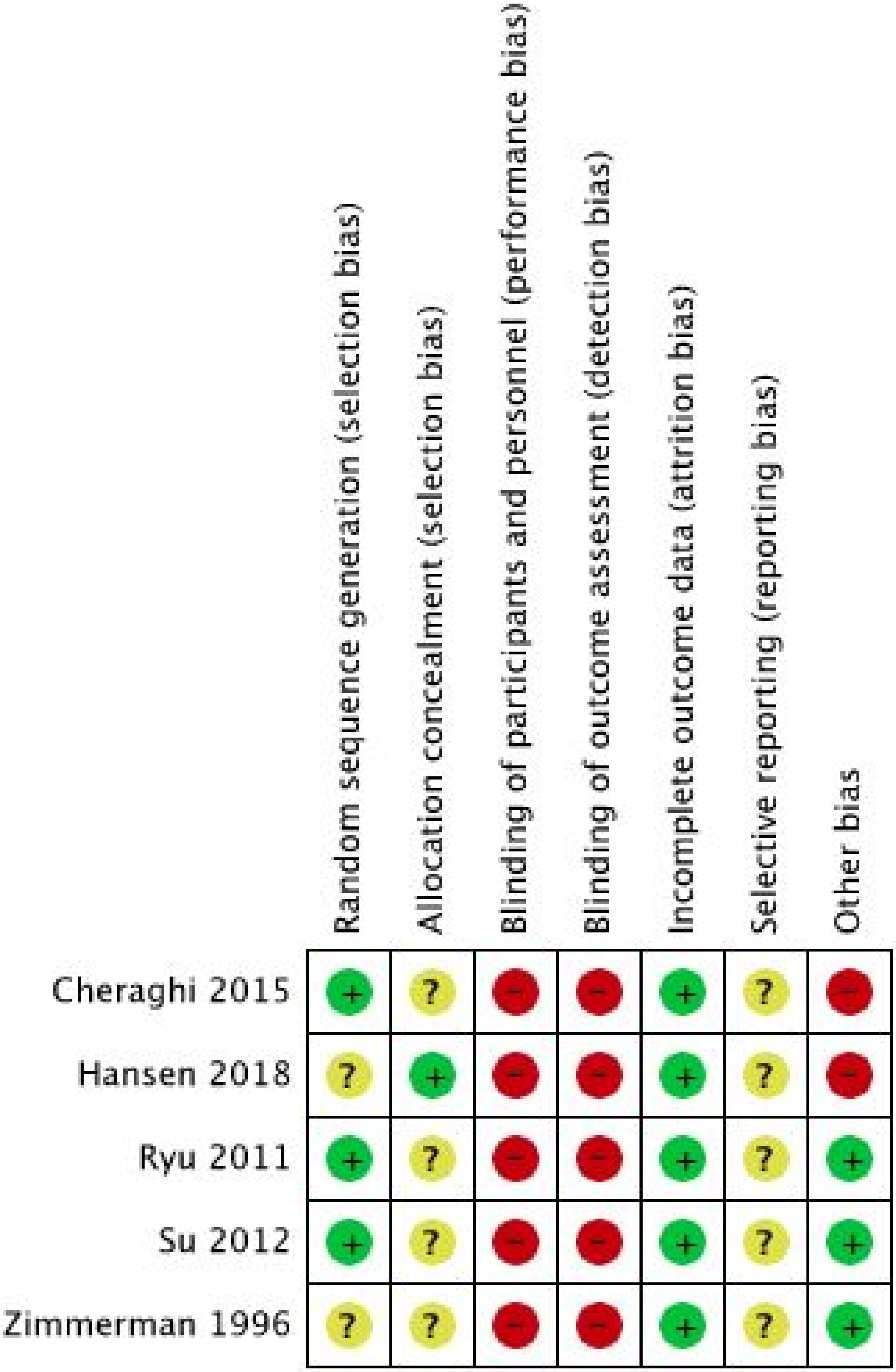

Figure 4. Summary of risk of bias

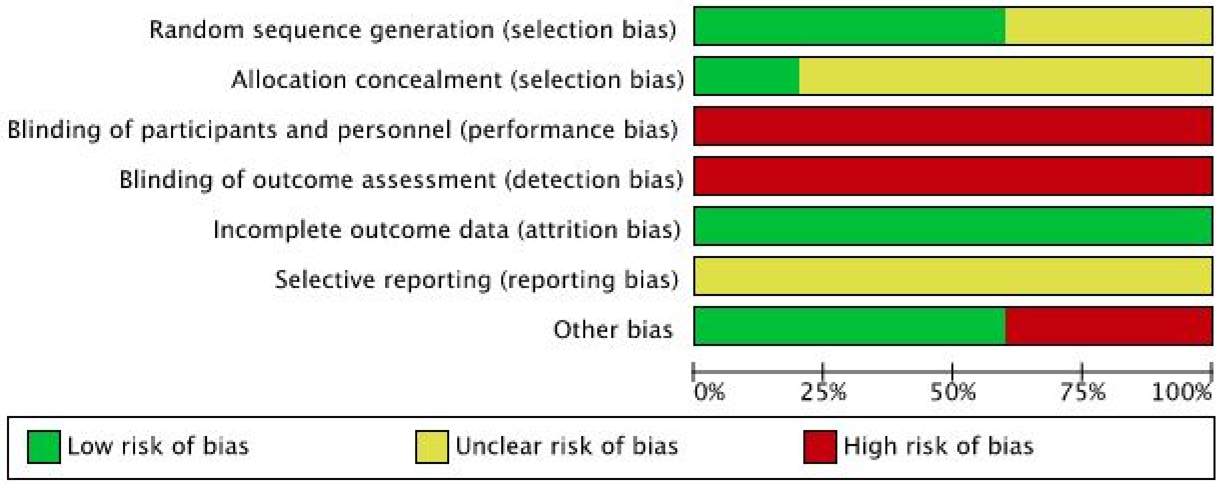

For peer review only

Supplementary file 1 PRISMA checklist

Section/topic	#	Checklist item	Reported on page #
**TITLE**			
Title	1	Identify the report as a systematic review, meta-analysis, or both.	1
**ABSTRACT**			
Structured summary	2	Provide a structured summary including, as applicable: background; objectives; data sources; study eligibility criteria, participants, and interventions; study appraisal and synthesis methods; results; limitations; conclusions and implications of key findings; systematic review registration number.	2/3
**INTRODUCTION**			
Rationale	3	Describe the rationale for the review in the context of what is already known.	4
Objectives	4	Provide an explicit statement of questions being addressed with reference to participants, interventions, comparisons, outcomes, and study design (PICOS).	5
**METHODS**			
Protocol and registration	5	Indicate if a review protocol exists, if and where it can be accessed (e.g., Web address), and, if available, provide registration information including registration number.	6
Eligibility criteria	6	Specify study characteristics (e.g., PICOS, length of follow-up) and report characteristics (e.g., years considered, language, publication status) used as criteria for eligibility, giving rationale.	6/7
Information sources	7	Describe all information sources (e.g., databases with dates of coverage, contact with study authors to identify additional studies) in the search and date last searched.	6
Search	8	Present full electronic search strategy for at least one database, including any limits used, such that it could be repeated.	Supplementary file 2
Study selection	9	State the process for selecting studies (i.e., screening, eligibility, included in systematic review, and, if applicable, included in the meta-analysis).	6/7
Data collection process	10	Describe method of data extraction from reports (e.g., piloted forms, independently, in duplicate) and any processes for obtaining and confirming data from investigators.	7
Data items	11	List and define all variables for which data were sought (e.g., PICOS, funding sources) and any assumptions and simplifications made.	7

For peer review only - http://bmjopen.bmj.com/site/about/guidelines.xhtml

Risk of bias in individual studies	12	Describe methods used for assessing risk of bias of individual studies (including specification of whether this was done at the study or outcome level), and how this information is to be used in any data synthesis.	7/8
Summary measures	13	State the principal summary measures (e.g., risk ratio, difference in means).	8
Synthesis of results	14	Describe the methods of handling data and combining results of studies, if done, including measures of consistency (e.g., I^2) for each meta-analysis.	8
Risk of bias across studies	15	Specify any assessment of risk of bias that may affect the cumulative evidence (e.g., publication bias, selective reporting within studies).	7/8
Additional analyses	16	Describe methods of additional analyses (e.g., sensitivity or subgroup analyses, meta-regression), if done, indicating which were pre-specified.	NA
**RESULTS**			
Study selection	17	Give numbers of studies screened, assessed for eligibility, and included in the review, with reasons for exclusions at each stage, ideally with a flow diagram.	9
Study characteristics	18	For each study, present characteristics for which data were extracted (e.g., study size, PICOS, follow-up period) and provide the citations.	9/10
Risk of bias within studies	19	Present data on risk of bias of each study and, if available, any outcome level assessment (see item 12).	11
Results of individual studies	20	For all outcomes considered (benefits or harms), present, for each study: (a) simple summary data for each intervention group (b) effect estimates and confidence intervals, ideally with a forest plot.	10/11
Synthesis of results	21	Present results of each meta-analysis done, including confidence intervals and measures of consistency.	10/11
Risk of bias across studies	22	Present results of any assessment of risk of bias across studies (see Item 15).	12
Additional analysis	23	Give results of additional analyses, if done (e.g., sensitivity or subgroup analyses, meta-regression [see Item 16]).	NA
**DISCUSSION**			
Summary of evidence	24	Summarize the main findings including the strength of evidence for each main outcome; consider their relevance to key groups (e.g., healthcare providers, users, and policy makers).	13
Limitations	25	Discuss limitations at study and outcome level (e.g., risk of bias), and at review-level (e.g., incomplete retrieval of identified research, reporting bias).	16/17
Conclusions	26	Provide a general interpretation of the results in the context of other evidence, and implications for future research.	17
**FUNDING**			
Funding	27	Describe sources of funding for the systematic review and other support (e.g., supply of data); role of funders for the systematic review.	17

For peer review only - http://bmjopen.bmj.com/site/about/guidelines.xhtml

Supplementary file 2 Search strategy

Database	Records identified	After de-duplication
embase.com	733	724
Medline Ovid	476	94
Web of science	457	187
Cochrane CENTRAL	261	53
Google scholar	200	121
Total	2127	1179

embase.com

(music/de OR 'music therapy'/de OR (music OR musical OR musicotherap*):ab,ti) AND ('sleep'/de OR 'night sleep'/de OR 'nonREM sleep'/de OR 'REM sleep'/de OR 'sleep induction'/de OR 'sleep pattern'/de OR 'sleep quality'/de OR 'sleep spindle'/de OR 'sleep stage'/de OR 'sleep time'/de OR 'sleep waking cycle'/de OR 'slow wave sleep'/de OR 'sleep disorder'/de OR 'insomnia'/de OR 'sleep parameters'/exp OR 'circadian rhythm'/de OR 'chronobiology'/de OR 'sleep hygiene'/de OR 'sleep deprivation'/de OR 'somnolence'/de OR 'sleep medicine'/exp OR (sleep* OR asleep* OR insomnia OR bedtime OR bed-time OR circadian* OR rem OR nonrem OR chronobiology* *OR somnolence**):ab,ti) NOT ([animals]/lim NOT [humans]/lim) NOT ([Conference Abstract]/lim) AND [English]/lim

Medline Ovid

(music/ OR "music therapy"/ OR (music OR musical OR musicotherap*).ab,ti.) AND (exp sleep/ OR Sleep Wake Disorders/ OR Sleep Deprivation/ OR Circadian Rhythm/ OR (sleep* OR asleep* OR insomnia OR bedtime OR bed-time OR circadian* OR rem OR nonrem OR chronobiology*).ab,ti.) NOT (exp animals/ NOT humans/) AND english.la.

For peer review only - http://bmjopen.bmj.com/site/about/guidelines.xhtml

Web of science

TS=(((music OR musical OR musicotherap*)) AND ((sleep* OR asleep* OR insomnia OR bedtime OR bed-time OR circadian* OR rem OR nonrem OR chronobiology*))) AND DT=(article) AND LA=(english)

Cochrane

((music OR musical OR musicotherap*):ab,ti) AND ((sleep* OR asleep* OR insomnia OR bedtime OR bed-time OR circadian* OR rem OR nonrem OR chronobiology*):ab,ti)

Google scholar

music|musical|musicotherapy sleep|asleep|insomnia|bedtime|"bed-time"|circadian|rem|nonrem|chronobiology

For peer review only

BMJ Open

Music intervention for sleep quality in critically ill and surgical patients: a meta-analysis

Journal:	*BMJ Open*
Manuscript ID	bmjopen-2020-042510.R1
Article Type:	Original research
Date Submitted by the Author:	19-Jan-2021
Complete List of Authors:	Kakar, Ellaha; Erasmus Medical Center, Neuroscience<br>Venema, Esmée; Maastricht University<br>Jeekel, Johannes; Erasmus MC<br>Klimek, M; Erasmus MC<br>van der Jagt, Mathieu; Erasmus MC, Department of Intensive Care
**Primary Subject Heading**:	Intensive care
Secondary Subject Heading:	Surgery
Keywords:	INTENSIVE & CRITICAL CARE, SURGERY, SLEEP MEDICINE

SCHOLARONE™
Manuscripts

I, the Submitting Author has the right to grant and does grant on behalf of all authors of the Work (as defined in the below author licence), an exclusive licence and/or a non-exclusive licence for contributions from authors who are: i) UK Crown employees; ii) where BMJ has agreed a CC-BY licence shall apply, and/or iii) in accordance with the terms applicable for US Federal Government officers or employees acting as part of their official duties; on a worldwide, perpetual, irrevocable, royalty-free basis to BMJ Publishing Group Ltd ("BMJ") its licensees and where the relevant Journal is co-owned by BMJ to the co-owners of the Journal, to publish the Work in this journal and any other BMJ products and to exploit all rights, as set out in our licence.

The Submitting Author accepts and understands that any supply made under these terms is made by BMJ to the Submitting Author unless you are acting as an employee on behalf of your employer or a postgraduate student of an affiliated institution which is paying any applicable article publishing charge ("APC") for Open Access articles. Where the Submitting Author wishes to make the Work available on an Open Access basis (and intends to pay the relevant APC), the terms of reuse of such Open Access shall be governed by a Creative Commons licence – details of these licences and which Creative Commons licence will apply to this Work are set out in our licence referred to above.

Other than as permitted in any relevant BMJ Author's Self Archiving Policies, I confirm this Work has not been accepted for publication elsewhere, is not being considered for publication elsewhere and does not duplicate material already published. I confirm all authors consent to publication of this Work and authorise the granting of this licence.

For peer review only

Music intervention for sleep quality in critically ill and surgical patients: a meta-analysis

E. Kakar, MD,[1,2*✉] E. Venema, BSc,[3*] J. Jeekel, MD, PhD,[1] M. Klimek, MD, PhD,[4] M. van der Jagt, MD, PhD[2]

*shared first authorship

✉ Corresponding Author; Miss Ellaha Kakar, address: Doctor Molewaterplein 40, 3015 GD Rotterdam Room NA-2123, phone: +31(0)10 704 35 41, email: e.kakar@erasmusmc.nl

[1] Department of Surgery, Erasmus MC, University Medical Center Rotterdam, the Netherlands

[2] Department of Intensive Care Unit, Erasmus MC, University Medical Center Rotterdam, the Netherlands

[3] Maastricht University, Maastricht, the Netherlands

[4] Department of Anesthesiology, Erasmus MC, University Medical Center Rotterdam, the Netherlands

For peer review only

For peer review only - http://bmjopen.bmj.com/site/about/guidelines.xhtml

Abstract

Objective Sleep disruption occurs frequently in hospitalized patients. Given the potential of music intervention as a non-pharmacological measure to improve sleep quality, we aimed to assess and quantify current literature on the effect of recorded music interventions on sleep quality and quantity in the adult critical care and surgical populations.

Design Systematic review and meta-analysis.

Data sources Embase, Medline Ovid, Cochrane Central, Web of Science, and Google Scholar.

Eligibility criteria for studies Randomized controlled trials assessing the effect of music on sleep quality in critically ill and surgical patients.

Methods The electronic databases were systematically searched from the 1st of January 1981 until the 27th of January 2020. Data were screened, extracted, and appraised by two independent reviewers. Primary outcomes were sleep quality and quantity, assessed with validated tools The PRISMA guidelines were followed. Random effects meta-analysis was performed, and pooled standardized mean differences (SMD) with 95% confidence intervals (CI) were reported.

Results Five studies (259 patients) were included in qualitative (risk of bias) and quantitative analysis (meta-analysis). Pooled data showed a significant effect of recorded music on subjective sleep quality in the critical care and surgical population (SMD =1.21 [95%CI 0.50; 1.91], p <0.01, excluding one non-English study; SMD =0.87 [95%CI 0.45; 1.29], p <0.01). The SMD of 1.21 corresponded to a 27.1% [95%CI 11.2; 42.8] increase in subjective sleep quality using validated questionnaires. A significant increase in subjective sleep quantity of 36 minutes was found in one study. Objective measurements of sleep assessed in one study using polysomnography, showed significant increase in deeper sleep stage in the music group.

Conclusions Recorded music showed a significant improvement in subjective sleep quality in some critical care and surgical populations. Therefore, its use may be relevant to improve sleep, but given the moderate potential for bias, further research is needed.

For peer review only

For peer review only - http://bmjopen.bmj.com/site/about/guidelines.xhtml

39 **PROSPERO registration number** CRD42020167783

40 **Keywords** Sleep, Music, Critical Care, Surgery

For peer review only

For peer review only - http://bmjopen.bmj.com/site/about/guidelines.xhtml

Article Summary

Strengths and limitations of this study

- This is the first comprehensive systematic review that studies the effect of music on sleep quality in critically ill and surgical patients.
- Patient reported outcomes, which are of increasing interest and of clinical relevance, are assessed in qualitative and quantitative analyses.
- Only data from randomized controlled trials are included.
- The risk of bias is moderate to high and the heterogeneity is high, which makes it difficult to draw definite conclusions.

For peer review only

For peer review only - http://bmjopen.bmj.com/site/about/guidelines.xhtml

Introduction

Sleep disruption is common in hospitalized patients.[1-4] A large cross-sectional study in the Netherlands found that sleep quality and quantity were significantly affected in hospitalized patients compared with patients' habitual sleep at home.[5] Sleep disruption is prevalent in almost all surgical and critically ill patients.[5-8] Important preventable factors associated with sleep disruption are pain, noise, anxiety, stress, sedative and analgesic medication usage, immobility, and severity of illness.[1, 5, 9-12]

In critically ill and surgical patients poor sleep has been associated with postoperative delirium, cardiovascular events in high-risk patients, post-operative fatigue (leading to prolonged immobility), altered mental status,[13-16] prolonged mechanical ventilation, cognitive impairment, altered immune function, and long term psychological comorbidities.[17] Thus, improving sleep promotes good health by improving emotional (anxiety, stress)[18] and possibly physical recovery. This is especially important for intensive care unit (ICU) and surgical patients given the loss of tissue integrity.[19, 20] Interventions in the hospital to improve sleep quality mainly include pharmacological interventions, such as benzodiazepines.[21-25] The Clinical Practice Guidelines for the Prevention and Management of Pain, Agitation/Sedation, Delirium, Immobility, and Sleep Disruption (PADIS) in Adult Patients in the ICU strongly recommend avoiding these drugs, given their potential to induce delirium and substance dependence.[17, 26, 27] Therefore, non-pharmacological measures are advised to prevent sleep disruption in hospitalized patients.[17, 28, 29 30-36]

Recently, several studies have shown a significant positive effect of perioperative music interventions on anxiety, pain, sedative and analgesic medication requirement, and neurohormonal stress response.[37-39] Also, in the ICU population associations have been made between music interventions and decreased serum cortisol levels, positive effects on state

For peer review only - http://bmjopen.bmj.com/site/about/guidelines.xhtml

anxiety, reduction in respiratory rate and systolic blood pressure, and decrease in sedative and analgesic requirement in mechanically ventilated patients.[40, 41]

Given the potential of music intervention as a non-pharmacological measure to improve sleep quality, we aimed to assess and quantify current literature on the effect of perioperative recorded music interventions on sleep quality and quantity in the adult critical care and surgical populations.

For peer review only

Materials and methods

Protocol and registration

This systematic review and meta-analysis follows the Preferred Reporting Items for Systematic Reviews and Meta-analysis (PRISMA) guidelines (supplementary file 1) and was registered in the PROSPERO database (https://www.crd.york.ac.uk/PROSPERO, as record number CRD42020167783).[42]

Search strategy

A systematic search was performed together with a dedicated biomedical information specialist in the Embase, Medline Ovid, Cochrane Central, Web of Science, and Google Scholar databases using a standardized protocol,[43] including articles between the 1st of January 1981 (the year in which the first minimally invasive surgical intervention was performed, in order to avoid old literature which is not compatible with the current standard perioperative pain practice) and 27th of January 2020. The search strategy included terminologies related to: sleep quality/insomnia, including sleep architecture (e.g. rapid eye movement [REM] sleep) and music (e.g. music therapy). The full search strategy per database is given in supplementary file 2.

Study screening and selection

Two reviewers (EK/EV) independently screened results of the search strategy on title/abstract, to confirm adherence to the eligibility criteria. Studies were eligible when they investigated (P) critically ill and/or surgical patients aged 18 years or older receiving a (I) recorded music intervention (C) compared to a control group in order to assess the effect on (O) sleep quality and quantity in (S) a randomized clinical trial (RCT). PICOS is a mnemonic used in evidence based medicine and stands for, respectively; Patient, Intervention, Control, Outcomes, and Study type.[44] Critically ill populations included patients admitted to ICU or cardiac care unit (CCU), since CCU patients were also considered to have (potential) vital organ failure. Other

For peer review only - http://bmjopen.bmj.com/site/about/guidelines.xhtml

eligibility criteria were; full text available and the study included in-hospital patients. Since, the above mentioned studies included patients who have the following in common; in-hospital patients and their compromised physiology (e.g. by performing an intervention, surgical procedure, or having critical illness), which have impact on the patients comfort and thus sleep, the data was suitable to be pooled. Studies involving live music performance were excluded, since live music performance consists of two interventions; the music and the interaction with the musician. Music intervention was defined as the use of recorded music consisting of melody, harmony, and rhythm. Nature sounds were only included when used in addition to music. If studies compared music to multiple groups, the group without music most similar to the music group was chosen as control group (e.g. if groups were 'scheduled rest' and 'standard care', 'scheduled rest' was chosen as control group if the music group also received a resting period). The data of the extra arms were therefore not used in this study. Full text articles were discussed for admissibility. All disagreements between reviewers were resolved by discussion.

Data collection process and items

Data were extracted and checked by the same two reviewers (EK/EV) independently according to a pre-designed dataset. The following study characteristics were extracted: author, year of publication, study type, country of study, reason for admission (e.g. surgery type), sample size, age (mean and standard deviation (SD)), gender (% male), severity of disease of included patients, type and timing of sleep assessment, timing of the intervention, setting (e.g. surgical ward, ICU, CCU), method of intervention delivery (e.g. headphones, CD-player), frequency and duration of the intervention, total duration of the intervention, music choice (e.g. patient- or researcher-selected), type of control group, and number of participants in the intervention and control group. Primary outcome measures were sleep quality and quantity measured after intervention or at the end of study period for both the control and intervention group. If a study

For peer review only - http://bmjopen.bmj.com/site/about/guidelines.xhtml

applied music intervention and collected data at multiple time points only the final time point was used, since music could have a cumulative effect.

Risk of bias assessment

Risk of bias within studies was assessed independently by two reviewers (EK/EV) using the Cochrane Risk of Bias tool for RCTs.[45] Incomplete data of ≥ 10% due to drop-out (attrition bias) was considered as high risk. If the study protocol was not available, the risk for selective reporting was considered as unclear. Publication bias was assessed by creating funnel plots and the risk of bias across studies of the effect of music on sleep quality was assessed using the GRADE (Grading of Recommendations, Assessment, Development and Evaluations) criteria.[45, 46]

Statistical analysis

Descriptive statistics were presented as means and their SD, counts (N), and percentages (%). For categorical age groups (e.g. age 20-50 = 8 patients, 51-60 = 7 patients) an approximation of the mean was calculated by taking the central point of the range as the mean for each category and pooling these means for each trial separately, weighted to the sample size. The overall % of males in this review was calculated by averaging the % of males weighted to the sample size. Age ranges of the samples were used to calculate approximations for the SD by calculation one-fourth of the range of the data ((maximum-minimum)/4).[47] Studies were included for quantitative analysis if mean values and SDs of the sleep scores were reported. The primary analysis included meta-analysis for sleep quality and quantity. Standardized mean differences (SMD) were calculated as summary statistics of the main outcome and a random effects model was used to calculate the overall effect of music on sleep quality and quantity, accounting for between-study heterogeneity. The between study-variance was estimated using the restricted maximum likelihood (REML) method. The level of heterogeneity was assessed using the I^2

For peer review only - http://bmjopen.bmj.com/site/about/guidelines.xhtml

statistic. For clinical interpretation the effect of the music intervention on sleep quality scores was expressed as a relative percentage increase, using a back-transformation of the acquired SMD that was described by the Cochrane Handbook for Systematic Reviews.[45] For this calculation, the SDs of the control groups were pooled to approximate the amount of variation in our patient population. Additionally the SD of a study with a large sample assessing sleep was used to make a more accurate approximation of the amount of variation. Data were analyzed using R version 3.6.3, and a two-sided p- value of < 0.05 was considered statistically significant. To calculate the increase in subjectively measured sleep quality based on the pooled SMD of the meta-analysis, a back-transformation was applied to the sleep quality as described by the Cochrane Handbook for Systematic Reviews.[45] For this back-transformation, the SDs of sleep were estimated by pooling the SDs of the control groups for the different assessment tools separately.

Patient and Public Involvement

No patients involved.

For peer review only

For peer review only - http://bmjopen.bmj.com/site/about/guidelines.xhtml

Results

The search yielded 2127 articles leaving 1179 articles after removal of duplicates, and 1146 articles after removal of articles before the year 1981. Titles and abstracts screening resulted in leaving ten articles for full text assessment. Five studies were excluded for the following reasons; full text not available, use of guided imaging in combination with music, use of nature sounds, and use of multiple interventions. Subsequently, a total of five RCTs[48-52] were included in the qualitative and quantitative analysis (259 patients) (Figure 1).

Study characteristics

A detailed overview of patient and music intervention characteristics is presented in Tables 1 and 2. Two studies were conducted at the ICU, two studies were conducted at the CCU and one study was performed in the surgical ward directly after ICU discharge. Apart from this study (Zimmerman et al.[52]) no other surgical papers were found. The mean age of the study population was 62.4 years with a predominance of males (65.8%). Hansen et al.[48] and Su et al.[51] conducted the study at the ICU. The study of Su et al.[51] included medical ICU patients, with an APACHE (acute physiology and chronic health evaluation) II score of ≤ 25, and the study of Hansen et al.[48] included surgical (59%) and medical (41%) ICU patients. Cheraghi et al.[49] and Ryu et al.[50] conducted their study at the CCU respectively including acute coronary syndrome (ACS) and percutaneous transluminal coronary angiography (PTCA) patients. The study of Zimmerman et al.[52] was performed in the nursing ward after coronary artery bypass graft (CABG) surgery. Since the abstract of the paper of Cheraghi et al.[49] was available in English, the study was included after title/abstract screening. The full text version was only available in the Persian language, the paper was translated by a statistician in the Erasmus MC with Persian as mother language. The study was included in this review due to acceptable methodology, which was not different than the other included studies.

For peer review only - http://bmjopen.bmj.com/site/about/guidelines.xhtml

Sleep quality was measured using the Richards-Campbell Sleep Questionnaire (RCSQ, 40%), the Verran and Snyder-Halpern sleep scale (VSH, 40%), and the Pittsburgh Sleep Quality Index (PSQI, 20%). Supplementary file 3 gives an overview of the previously mentioned sleep questionnaire characteristics. Only in the study of Ryu et al. sleep quantity was measured using patient questionnaires. In the study of Su et al. sleep quality and quantity was also measured objectively, using the polysomnography (PSG).[51] PSG is currently the golden standard in objectively measuring sleep variables, including total sleep time (TST), wake after sleep onset (WASO), sleep onset latency (SOL), REM latency, sleep efficiency (SE), arousal index (AI), and percentage of total sleep time spent in each sleep stage (N1, N2, N3, and REM).[53]

For peer review only

For peer review only - http://bmjopen.bmj.com/site/about/guidelines.xhtml

Table 1 Study characteristics

Study	Study type	Country	Reason for admission	Sample size	Mean age (SD)		Gender (% male)		Outcome assessment	Severity of disease of included patients
					Intervention	Control	Intervention	Control		
**[1]Cheraghi, 2015**	RCT	Iran	ACS	72	56.9(10.5)	62.1(12.7)	80.6%	80.6%	Sleep quality (PSQI)	- Oriented in person, time, and place<br>- Hemodynamically stable<br>- No ventilation<br>- History of hospitalization; 55.6%
**[2]Hansen, 2018**	RCT	Denmark	Surgical and medical ICU	37	60.0(18.0)	65(16.0)	55.6%	52.6%	Sleep quality (RCSQ)	- GCS; ≥ 14<br>- Communicable<br>- ICU LOS; 3 (3)<br>- Comorbidity; 59.0%<br>- Planned hospitalization; 49.0%
**[3]Ryu, 2011**	RCT	South Korea	PTCA	58	58.5(15.0)	59.9(15.0)	65.5%	65.5%	Sleep quality (VSH), sleep quantity (questionnaire)	- No ventilation<br>- No use of sleep inducing or sedative medications
**[4]Su, 2012**	RCT	Taiwan	Medical ICU	28	62.4(9.1)	60.9(10.8)	57.1%	64.3%	Sleep quality (VSH and PSG)	- Communicable<br>- Hemodynamically stable<br>- ICU stay; ≥ 24 hours<br>- Free from symptoms of current infection.<br>- APACHE II; 18.6 (3.8)<br>- On ventilation; 35.7%
**[5]Zimmerman, 1996**	RCT	USA	CABG	64	67.0(9.9)	67.0(9.9)	68.0%	68.0%	Sleep quality (RCSQ)	- Oriented in person, time, and place<br>- Extubated within 24 hours<br>- Hospital LOS 5.8 (2.3) days<br>- Previous surgery; 90.0%
**Summary of all studies**				259	60.8(12.6)	63.1(11.2)	68.0%	64.4%		

SD = standard deviation; RCT = randomized controlled trial; ICU = intensive care unit; ACS = acute coronary syndrome; PTCA = percutaneous transluminal coronary angioplasty; CABG = coronary artery bypass graft; PSQI = Pittsburgh sleep quality index; RCSQ = Richards-Campbell sleep questionnaire; VSH = Verran and Snyder-Halpern sleep scale; PSG = polysomnography; USA = United States of America; GCS = Glasgow Coma Scale; LOS = Length Of Stay; APACHE = Acute Physiology And Chronic Health Evaluation. Data are reported as means (standard deviations) and percentages (%).

[1]Mali Cheraghi, et al. The effect of instrumental music on sleep in patients admitted to Coronary Care Unit: sid.ir; 2015.

[2]Hansen IP, et al. Effects of music during daytime rest in the intensive care unit. Nurs Crit Care. 2018;23(4):207-13.

[3]Ryu MJ, et al. Effect of sleep-inducing music on sleep in persons with percutaneous transluminal coronary angiography in the cardiac care unit. J Clin Nurs. 2012;21(5-6):728-35.

[4]Su CP, et al. A randomized controlled trial of the effects of listening to non-commercial music on quality of nocturnal sleep and relaxation indices in patients in medical intensive care unit. J Adv Nurs. 2013;69(6):1377-89.

[5]Zimmerman L, et al. The effects of music interventions on postoperative pain and sleep in coronary artery bypass graft (CABG) patients. Sch Inq Nurs Pract. 1996;10(2):153-70; discussion 71-4.

For peer review only - http://bmjopen.bmj.com/site/about/guidelines.xhtml

Table 2 Music intervention characteristics

Study	Location	N music group	Intervention	Intervention choice	Medium	Frequency (per day) x duration (min.)	Total duration (min.)	Timing intervention	Control	N control group	Timing sleep assessment
**Cheraghi, 2015**	CCU	36	Non-vocal music, accepted by the society	Researcher	Headphones	3 x 45	135	Every night after admission during 3 nights before sleep	Standard care	36	Baseline and every morning during three consecutive days
**Hansen, 2018**	ICU	18	MusiCure: soothing music, soft wind, bird twitter, ocean sound, music instruments	Researcher	Loudspeaker in the ceiling of patients' bed	1 x 30	30	During the day on day 3 after admission	Standard care during rest	19	Once directly after intervention
**Ryu, 2011**	CCU	29	Sleep inducing music: nature sounds, delta wave control music, and Goldberg variations BWV + eye bandages	Researcher	Earphones	1 x 53	53	Postoperative in the evening, timing NR	No music with earplugs and eye-shield	29	Once in the morning after intervention
**Su, 2012**	ICU	14	Sedating piano music: smooth rhythm to achieve a relaxing effect, a tempo of 60-80 beats/min, minor tonalities, smooth melody lines and no dramatic changes in volume and rhythm	Researcher	CD player	1 x 45	45	Night of day 3 after admission	Same procedure, no music	14	Baseline VSH and PSG, PSG first 2 hours of night of intervention, VSH once in the morning after intervention
**Zimmerman, 1996**	Ward	32	Country western instrumental, fresh aire (Mannheim Steamroller), winter into spring (George Winston), prelude and comfort zone (Steven Halpern) (facilitates relaxation)	Option out of 5 audiotapes, in case of no preference Halpern was chosen	Headphones	2 x 30	60	Afternoon or early evening on POD 2-3	Scheduled rest	32	Baseline and once in the morning on POD 3

ICU = intensive care unit; CCU = coronary care unit; POD = postoperative day; NR = not reported; VSH = Verran and Snyder-Halpern sleep scale; PSG = polysomnography.

For peer review only - http://bmjopen.bmj.com/site/about/guidelines.xhtml

Music was mostly described as being soothing, sleep inducing, or sedating (60%).[48, 50, 51] Music was selected by the researcher in four of the five studies (80%), only Zimmerman et al. provided the option of choice between five audiotapes according to the patients' preference (Table 2).[52] Music was mostly administered using head- or earphones (60%). Duration of the intervention was on average 40 minutes per session and ranged from 30 to 53 minutes per day. Cheraghi et al.[49] administered the music intervention during three consecutive evenings, just before bedtime, after admission to the CCU, and assessed sleep at baseline and every morning after an evening of intervention. In the study of Su et al.[51] the intervention was only administered in the night of day three after admission to the ICU and sleep was assessed at baseline (PSG and VSH) and PSG during the first 2 hours of sleep on the same night and VSH in the morning after the night of intervention. Hansen et al. applied the intervention during the day on day three after admission and assessed sleep once immediately afterwards. Zimmerman et al.[52] applied the intervention on the night of postoperative day (POD) two and assessed sleep at baseline and the morning of POD three. Ryu et al.[50] did not specify on which POD the music intervention and sleep assessment was performed. Baseline sleep assessment was carried out in only three studies,[49, 51, 52] no differences were found between the study groups in any of the studies based on baseline data. Control groups received either standard care (2 studies), scheduled rest (2 studies), or standard care with earplugs and eye shields (1 study).

Effect of music on subjectively measured sleep quality

Sleep quality scores of the final music session were pooled.[48-52] For the data of Cheraghi et al.[49] we applied reverse scoring since they used the PSQI, which was the only tool assessing sleep quality as better when scored lower on the scale. Pooling data resulted in an overall significant effect of recorded music on sleep quality in critical care and surgical patients (SMD= 1.21 [95% CI 0.50; 1.91], $p < 0.01$). Figure 2A/B presents the forest and funnel plot. The study of Cheraghi et al. showed a relative large effect compared to the other studies. Pooled data without the

study of Cheraghi et al. the effect remained significant (SMD = 0.87 [95% CI 0.45; 1.29], $p < 0.01$, Figure 2C).

Since out meta-analysis eventually only included subjectively measures sleep assessment tools, the back-transformation was based on a recent Dutch study which assessed sleep in 194 patient using the RCSQ, which is a validated and reliable sleep quality assessment tool in the critically ill.[54] The reported median inter quartile range (IQR) for the RCSQ reported in this study was transformed to mean ± SD using the methodology of Wan et al.[47] This resulted in an SD approximation of 2.24. Using the effect size of our meta- analysis, SMD = 1.21, this resulted in a reduction of 2.71 [95% CI 1.12; 4.28] on the RCSQ which ranges from zero to ten. This implies a 27.1% [95% CI 11.2; 42.8] increase in sleep quality in the ICU due to a music intervention. In order to check if the effect in our study population approximates the effect calculated above we also applied the back-transformation using the pooled RCSQ SDs (N = 51, SD = 2.53) of the control groups in our study. This resulted in a reduction of 3.06 [95% CI 1.27; 4.83] on the RCSQ, equal to 30.6% [95% CI 12.7; 48.3] increase in sleep quality.

For peer review only

Effect of music on subjectively measured sleep quantity

Meta-analysis on the effect of music on sleep quantity could not be performed since it was only assessed by the study of Ryu et al in PTCA patients.[50] This study found a statistically significant higher sleep duration in the music group (N = 29) compared to the control group (N = 29); 279.31 ± 43.99 respectively 243.10 ± 42.68 with a p- value of 0.002.

Effect of music on objectively measured sleep

As mentioned before, only the study of Su et al. measured sleep objectively using the PSG. In this study they found a significant difference in the N2 (Wald $\square^2 = 6.03$, $p = 0.014$) and N3 (Wald

For peer review only - http://bmjopen.bmj.com/site/about/guidelines.xhtml

□[2] = 7.02, p = 0.008) sleep stages, indicating that the music group had a shorter N2 sleep stage and a longer N3 sleep stage.

Risk of bias assessment

The overall risk of bias was moderate to high (Figure 3). All included studies reported the use of randomization, but a high risk of selection bias was considered due to insufficient details regarding random sequence generation in two studies,[48, 52] and allocation concealment in four studies.[49-52] Due to the type of intervention and the subjective outcome assessment blinding of participants and personnel was not possible, therefore performance and detection bias were considered high in all studies. Attrition bias was considered low in all studies since none of the studies had a drop-out rate of ≥ 10%. A full study protocol was missing (assessed by checking reported registration number of the trial in the paper and registers for clinical trials (e.g. clinicaltrials.gov) for all included studies leading to an unclear risk for reporting bias. Two included studies (20%) had a high risk of bias due to other reasons; Cheraghi et al.[49] excluded patients if listening to the music induced anxiety or brought up bad memories, which could have contributed to an overestimation of the effect of music intervention on sleep quality, and Hansen et al.[48] measured sleep quality during daytime rest with the RCSQ which is only a validated measurement tool for sleep quality after nocturnal sleep. Figure 4 presents the summary of the risk of bias assessment.

Grade certainty rating

Risk of bias was moderate to high for sleep quality. Imprecision was considered medium since the effect size in the 95% CI of the pooled estimate ranged from medium to high, which could influence clinical decision-making. Consistency was considered moderate to high since individual studies included in the meta-analysis showed a positive influence of music on sleep quality, the 95% CIs overlapped, but the heterogeneity was high. Heterogeneity was moderate

For peer review only - http://bmjopen.bmj.com/site/about/guidelines.xhtml

291 to high and statistically significant for sleep quality (I^2 = 85% [95% CI 68%; 93%, T^2 = 0.5, $p < 0.01$), possibly caused by the differences in patient characteristics. After the exclusion of the outlier study of Cheraghi et al. from the meta-analysis, heterogeneity decreased and was non-significant (I^2 = 45% CI 0%; 82%, T^2 = 0.08, p = 0.14). Meta-regression, to assess the reason for high heterogeneity, could not be performed since there was a limited number of papers available. Directness was considered high as studies directly investigated music in the population of interest, the intervention was mostly applied on the third day after ICU/CCU admission or after surgery, and the reported outcomes were critical for decision-making (e.g. if sleep quality was low, administration of sleep medication could be considered). Scattering in the funnel plot is symmetrical, therefore the risk for publication bias is considered low. In conclusion, we rate the GRADE certainty rating as moderate.

For peer review only

Discussion

This meta-analysis showed a significant beneficial effect of recorded music intervention on subjectively measures of sleep quality in critically ill and post-CABG patients admitted to the ward (SMD= 1.21 [95% CI 0.50; 1.91], $p < 0.01$), when on average 40 minutes (range 30 – 53) per session/day of music is applied. The overall risk of bias was moderate to high.

These findings are in line with the current literature.[29, 55-57] Hu et al. reviewed literature for the effect of non-pharmacological interventions on sleep quality in the ICU.[29] Although, they could not pool data and their quality of evidence was low, they found that music intervention might positively influence sleep quality. Also, Feng et al. studied the effect of music and other non-pharmacological interventions (e.g. music combined with other interventions, acupuncture) on primary insomnia (insomnia after ruling out several other conditions such as psychiatric (depression and anxiety), medical (pain), circadian (phase-delay syndrome), or other sleep disorders. They found that solely music intervention had the highest ranking and seemed to offer clear advantages on sleep quality.[55] Our findings are also in line with the current literature on the effect of music on other outcomes in the critically ill population. These studies show effectiveness of music on anxiety, pain, vital parameters, inflammatory markers, and medication requirement.[40, 41, 58-60]

Sleep architecture in critical care patients is characterized by decreased total sleep time (TST), sleep divided into short discrete episodes, sleep occurring mostly during daytime, increased amount of light sleep stages and decreased amount of deeper sleep stage.[8, 17, 61-64] This leads to loss of the restorative and resting function of sleep, which is achieved by deeper sleep stages.[40, 61, 63, 65] Sleep disturbances have been associated with cognitive impairment, including delirium, prolonged duration of mechanical ventilation, altered immune function, and long-term psychological comorbidities.[8, 17, 66-69] Furthermore, acutely developed sleep disturbances in the

For peer review only - http://bmjopen.bmj.com/site/about/guidelines.xhtml

ICU can persist after discharge and is the most common stressful factor experienced by patients.[61, 70-73] Further, the administration of sedative and analgesic medication, aimed to improve patient comfort, may negatively impact sleep quality.[5, 10-12, 74-78] Therefore, preventing sleep disturbances in critically ill patients is highly important . The one study in our review that assessed sleep objectively found that music significantly increased the deeper sleep stage N3 and decreased the light sleep stage N2, inevitably strengthening the results of our meta-analysis.[51]

We made an approximation of 27.1% increase in sleep quality if music intervention is applied based on our pooled data. Several studies comparing benzodiazepines to a placebo showed even lower efficacy, ranging from 12.9% to 21.4% improvement. [79] [80] [81] [82] This indicates that music may have a similar or even better effect on sleep quality compared with drug therapy with benzodiazepines, while the patient is not exposed to their harmful side-effects and risk of dependency. Therefore, the use of recorded music in hospitalized patients can be considered a clinically relevant intervention. Only one paper in this study assessed effect of music on sleep quantity and found a significant increase in sleep duration of 36 min. in a sample of 58 patients.[50] Krenk et al. found that Zolpidem shortened TST with 28 min. compared to the placebo group, this finding was not significant.[81] Simons et al. found a significant extension of 57 min. when using Temazepam in healthy volunteers.[82] Also, Sharma et al. measured an increased TST when Zolpidem was used, unfortunately effects on deeper sleep stages were not found. Of not, extension of the TST does not necessarily lead to better sleep, since it depends more on restorative sleep.

The physiology behind the effect of music on sleep remains unclear. Several theories have been proposed on the effect of music on sleep including; rhythmic entrainment, masking, and distraction.[57] It is important to recognize that the effect of music also entails the neuro- and psychophysiological levels.[83-86] Literature indicates that music induces the anxiolytic effect

For peer review only - http://bmjopen.bmj.com/site/about/guidelines.xhtml

through suppression of the sympathetic nervous system.[87, 88] Also, music stimulates the release of endorphins by activating memory and the limbic system, which plays an important role in emotional well-being.[58, 89] More specifically, the nucleus accumbens is activated leading to increased dopamine release and deactivation of areas in the brain related to stress and cortisol signaling.[90] Furthermore, several studies have suggested positive effects of vagus nerve stimulation (VNS)[91] on objective and subjective sleep parameters in epilepsy patients[92, 93]. Unfortunately, in spite of these theoretical considerations, we did not find literature on the mechanistic effect of music on sleep architecture. Thus, sleep disturbances are multifactorial, and non-pharmacological interventions should focus on tackling multiple factors (anxiety, pain, stress etc.) affecting sleep at the same time.

The GRADE certainty was rated moderate, which supports the estimated clinical relevant effects. Unfortunately, the relatively small number of studies and sample sizes lead to uncertain reliability and validity of this study, which is also seen in the current literature regarding the effect of music on sleep in other populations.[79-81]

Strengths and limitations

The sleep assessment tools used in the included studies of this meta-analysis were all validated, reliable, and easily applicable. All tools used in the studies included in the meta-analysis are validated and reliable self-reporting questionnaires assessing sleep health of the previous night, and are used to assess sleep quality in the studies included in this meta-analysis. The tools are widely used to measure sleep quality in hospitalized patients, and thus are deemed conceptually to perform meta-analysis.[94] An important limitation of this review is the limited amount of studies included for quantitative analysis. Second, heterogeneity was high. Third, there was a moderate to high risk of bias, due to insufficient information on random sequence generation, allocation concealment, and because the primary outcome included

For peer review only - http://bmjopen.bmj.com/site/about/guidelines.xhtml

subjective patient reported questionnaires and blinding of participants in a music intervention study is not feasible, although Chlan et al. (2013) in a large clinical trial on music intervention, that found a decrease in anxiety in the music group, tried to avoid bias due to non-blinding by including an extra control group wearing noise-cancelling headphones (without music).

Future research recommendations

We suggest future research should consist of high quality randomized clinical trials with the use of objective tools for sleep assessment, as recorded music seems effective and clinically relevant, in order to make more definite conclusions regarding the effect of recorded music on sleep. Since, until now relatively small studies with a high variability in the music "dose" are conducted we recommend future studies to focus on larger sample sizes with a high methodological quality in order to avoid a substantial risk of bias. Studies with music interventions should report the type of music, timing, duration, and frequency of the intervention and sleep assessment in their studies with a validated and reproducible tool. Our analyses suggest that a minimum of 30 minutes per day/session is sufficient in order for the music intervention to be effective for the sleep quality. This minimum of 30 minutes of music per session/day is also consistent with the current literature.[37, 40, 59] Previous studies can serve as a guide for future studies.[95]

Conclusion

This systematic review and meta-analysis of randomized controlled trials showed that recorded music interventions significantly increase sleep quality in the critically ill, ACS or PTCA patients and after cardiac surgery for coronary artery disease. Music is easily applicable and has no risks and side-effects and should therefore be considered as a suitable non-medicinal alternative for sleep quality improvement in these patient groups. Since the clinical trials

For peer review only - http://bmjopen.bmj.com/site/about/guidelines.xhtml

performed until now are small and of low quality, we suggest larger and high quality randomized clinical trials for future research, including broader patient populations.

Acknowledgements

The authors thank W. Bramer, biomedical information specialist of the Medical Library at the Erasmus University Medical Centre, for his assistance in the literature search and J. van Rosmalen, assistant professor at the Department of Biostatistics of the Erasmus University Medical Centre, for his assistance in the statistical analysis.

Funding This research received no specific grant from any funding agency in the public, commercial or not-for-profit sectors.

Disclosure The authors declare no other conflict of interest.

Contributorship statement JJ conceived the study idea. EK and EV coordinated the systematic review. EK and EV screened abstracts and full texts. EK and EV wrote the first draft of the manuscript and judged risk of bias in the studies. EK, EV, MJ, MK, and JJ interpreted the data. EK, EV, MJ, MK, and JJ critically revised the manuscript. EK, EV, MJ, MK, and JJ had full access to all of the data in the study and can take responsibility for the integrity of the data and the accuracy of the data analysis.

Data availability Data sharing not applicable as no datasets generated and/or analyzed for this study.

For peer review only - http://bmjopen.bmj.com/site/about/guidelines.xhtml

Figure legend

Figure 1. PRISMA Flor Diagram. N indicates the number of articles.

Figure 2A. Forest plot assessing the effect of music on subjective sleep quality.

Figure 2B. Funnel plot assessing the effect of music on subjective sleep quality.

Figure 2C. Forest plot assessing the effect of music on subjective sleep, excluding the paper of Cheraghi et al.

Figure 3. Risk of bias assessment.

Figure 4. Summary of risk of bias.

For peer review only

For peer review only - http://bmjopen.bmj.com/site/about/guidelines.xhtml

References

1. Dobing S, et al. Sleep Quality and Factors Influencing Self-Reported Sleep Duration and Quality in the General Internal Medicine Inpatient Population. PLoS One. 2016;11(6):e0156735.
2. Dogan O, et al. Sleep quality in hospitalized patients. J Clin Nurs. 2005;14(1):107-13.
3. Jakobsen G, et al. Sleep quality in hospitalized patients with advanced cancer: an observational study using self-reports of sleep and actigraphy. Support Care Cancer. 2020;28(4):2015-23.
4. Park MJ, Kim KH. What affects the subjective sleep quality of hospitalized elderly patients? Geriatr Gerontol Int. 2017;17(3):471-9.
5. Wesselius HM, et al. Quality and Quantity of Sleep and Factors Associated With Sleep Disturbance in Hospitalized Patients. JAMA Intern Med. 2018;178(9):1201-8.
6. Cilingir D, et al. Factors affecting the sleep status of surgical and medical patients at a University Hospital of Turkey. J Pak Med Assoc. 2016;66(12):1535-40.
7. Bani Younis M, Hayajneh FA. Quality of Sleep Among Intensive Care Unit Patients: A Literature Review. Crit Care Nurs Q. 2018;41(2):170-7.
8. Pulak LM, Jensen L. Sleep in the Intensive Care Unit: A Review. J Intensive Care Med. 2016;31(1):14-23.
9. Parthasarathy S, et al. Biological validity to sleep measurements during critical illness. Crit Care Med. 2010;38(2):705-6.
10. DeMartinis NA, et al. New approaches for the treatment of sleep disorders. Adv Pharmacol. 2009;57:187-235.
11. Yilmaz M, et al. Sleep quality of hospitalized patients in surgical units. Nurs Forum. 2012;47(3):183-92.
12. Klemann N, et al. Factors affecting post-operative sleep in patients undergoing colorectal surgery - a systematic review. Dan Med J. 2015;62(4):A5053.
13. Su X, Wang DX. Improve postoperative sleep: what can we do? Curr Opin Anaesthesiol. 2018;31(1):83-8.
14. Kjolhede P, et al. The impact of quality of sleep on recovery from fast-track abdominal hysterectomy. J Clin Sleep Med. 2012;8(4):395-402.
15. Rosenberg J. Sleep disturbances after non-cardiac surgery. Sleep Med Rev. 2001;5(2):129-37.
16. Gogenur I. Postoperative circadian disturbances. Dan Med Bull. 2010;57(12):B4205.
17. Devlin JW, et al. Clinical Practice Guidelines for the Prevention and Management of Pain, Agitation/Sedation, Delirium, Immobility, and Sleep Disruption in Adult Patients in the ICU. Crit Care Med. 2018;46(9):e825-e73.
18. Lieberman HR, et al. Severe decrements in cognition function and mood induced by sleep loss, heat, dehydration, and undernutrition during simulated combat. Biol Psychiatry. 2005;57(4):422-9.
19. Telias I, Wilcox ME. Sleep and Circadian Rhythm in Critical Illness. Crit Care. 2019;23(1):82.
20. Peever J, Fuller PM. The Biology of REM Sleep. Curr Biol. 2017;27(22):R1237-R48.
21. Gillis CM, et al. Inpatient pharmacological sleep aid utilization is common at a tertiary medical center. J Hosp Med. 2014;9(10):652-7.
22. Frighetto L, et al. An assessment of quality of sleep and the use of drugs with sedating properties in hospitalized adult patients. Health Qual Life Outcomes. 2004;2:17.
23. van Beek S, et al. The effect of midazolam as premedication on the quality of postoperative recovery after laparotomy: a randomized clinical trial
Effet du midazolam en premedication sur la qualite de la recuperation postoperatoire apres une laparotomie : une etude clinique randomisee. Can J Anaesth. 2020;67(1):32-41.

For peer review only

For peer review only - http://bmjopen.bmj.com/site/about/guidelines.xhtml

24. Maurice-Szamburski A, et al. Effect of sedative premedication on patient experience after general anesthesia: a randomized clinical trial. JAMA. 2015;313(9):916-25.
25. Mijderwijk H, et al. Lorazepam does not improve the quality of recovery in day-case surgery patients: a randomised placebo-controlled clinical trial. Eur J Anaesthesiol. 2013;30(12):743-51.
26. Halfens R, et al. Effect of the use of sleep medication in Dutch hospitals on the use of sleep medication at home. J Adv Nurs. 1994;19(1):66-70.
27. Brummett CM, et al. New Persistent Opioid Use After Minor and Major Surgical Procedures in US Adults. JAMA Surg. 2017;152(6):e170504.
28. Lenhart SE, Buysse DJ. Treatment of insomnia in hospitalized patients. Ann Pharmacother. 2001;35(11):1449-57.
29. Hu RF, et al. Non-pharmacological interventions for sleep promotion in the intensive care unit. Cochrane Database Syst Rev. 2015(10):CD008808.
30. Brandt J, Leong C. Benzodiazepines and Z-Drugs: An Updated Review of Major Adverse Outcomes Reported on in Epidemiologic Research. Drugs R D. 2017;17(4):493-507.
31. Glass J, et al. Sedative hypnotics in older people with insomnia: meta-analysis of risks and benefits. BMJ. 2005;331(7526):1169.
32. Weinbroum AA, et al. The midazolam-induced paradox phenomenon is reversible by flumazenil. Epidemiology, patient characteristics and review of the literature. Eur J Anaesthesiol. 2001;18(12):789-97.
33. American Geriatrics Society Expert Panel on Postoperative Delirium in Older A. American Geriatrics Society abstracted clinical practice guideline for postoperative delirium in older adults. J Am Geriatr Soc. 2015;63(1):142-50.
34. Bringman H, et al. Relaxing music as pre-medication before surgery: a randomised controlled trial. Acta Anaesthesiol Scand. 2009;53(6):759-64.
35. Kassie GM, et al. Preoperative medication use and postoperative delirium: a systematic review. BMC Geriatr. 2017;17(1):298.
36. Zaal IJ, et al. Benzodiazepine-associated delirium in critically ill adults. Intensive Care Med. 2015;41(12):2130-7.
37. Fu VX, et al. The Effect of Perioperative Music on Medication Requirement and Hospital Length of Stay: A Meta-analysis. Ann Surg. 2019.
38. Fu VX, et al. The Effect of Perioperative Music on the Stress Response to Surgery: A Meta-analysis. J Surg Res. 2019;244:444-55.
39. Kuhlmann AYR, et al. Meta-analysis evaluating music interventions for anxiety and pain in surgery. Br J Surg. 2018;105(7):773-83.
40. Bradt J, Dileo C. Music interventions for mechanically ventilated patients. Cochrane Database Syst Rev. 2014(12):CD006902.
41. Khan SH, et al. Effects of music intervention on inflammatory markers in critically ill and post-operative patients: A systematic review of the literature. Heart Lung. 2018;47(5):489-96.
42. Moher D, et al. Preferred reporting items for systematic reviews and meta-analyses: the PRISMA statement. PLoS Med. 2009;6(7):e1000097.
43. Bramer WM, et al. Evaluation of a new method for librarian-mediated literature searches for systematic reviews. Res Synth Methods. 2018;9(4):510-20.
44. Schardt C, et al. Utilization of the PICO framework to improve searching PubMed for clinical questions. BMC Med Inform Decis Mak. 2007;7:16.
45. Higgins JPT GS. The Cochrane Handbook for Systematic Reviews of Interventions Version 5.1.0 2011.
46. Guyatt GH, et al. GRADE: an emerging consensus on rating quality of evidence and strength of recommendations. BMJ. 2008;336(7650):924-6.
47. Wan X, et al. Estimating the sample mean and standard deviation from the sample size, median, range and/or interquartile range. BMC Med Res Methodol. 2014;14:135.

For peer review only

For peer review only - http://bmjopen.bmj.com/site/about/guidelines.xhtml

48. Hansen IP, et al. Effects of music during daytime rest in the intensive care unit. Nurs Crit Care. 2018;23(4):207-13.
49. Mali C, et al. The effect of instrumental music on sleep in patients admitted to Coronary Care Unit: sid.ir; 2015.
50. Ryu MJ, et al. Effect of sleep-inducing music on sleep in persons with percutaneous transluminal coronary angiography in the cardiac care unit. J Clin Nurs. 2012;21(5-6):728-35.
51. Su CP, et al. A randomized controlled trial of the effects of listening to non-commercial music on quality of nocturnal sleep and relaxation indices in patients in medical intensive care unit. J Adv Nurs. 2013;69(6):1377-89.
52. Zimmerman L, et al. The effects of music interventions on postoperative pain and sleep in coronary artery bypass graft (CABG) patients. Sch Inq Nurs Pract. 1996;10(2):153-70; discussion 71-4.
53. Morgenthaler TI, et al. Practice parameters for the clinical evaluation and treatment of circadian rhythm sleep disorders. An American Academy of Sleep Medicine report. Sleep. 2007;30(11):1445-59.
54. Rood P, et al. Development and daily use of a numeric rating score to assess sleep quality in ICU patients. J Crit Care. 2019;52:68-74.
55. Feng F, et al. Can music improve sleep quality in adults with primary insomnia? A systematic review and network meta-analysis. Int J Nurs Stud. 2018;77:189-96.
56. Bani Younis M, et al. Measurement and Nonpharmacologic Management of Sleep Disturbance in the Intensive Care Units: A Literature Review. Crit Care Nurs Q. 2019;42(1):75-80.
57. Dickson GT, Schubert E. How does music aid sleep? literature review. Sleep Med. 2019;63:142-50.
58. Beaulieu-Boire G, et al. Music and biological stress dampening in mechanically-ventilated patients at the intensive care unit ward-a prospective interventional randomized crossover trial. J Crit Care. 2013;28(4):442-50.
59. Richard-Lalonde M, et al. The Effect of Music on Pain in the Adult Intensive Care Unit: A Systematic Review of Randomized Controlled Trials. J Pain Symptom Manage. 2020;59(6):1304-19 e6.
60. Umbrello M, et al. Music therapy reduces stress and anxiety in critically ill patients: a systematic review of randomized clinical trials. Minerva Anestesiol. 2019;85(8):886-98.
61. Pisani MA, et al. Sleep in the intensive care unit. Am J Respir Crit Care Med. 2015;191(7):731-8.
62. Cooper AB, et al. Sleep in critically ill patients requiring mechanical ventilation. Chest. 2000;117(3):809-18.
63. Freedman NS, et al. Abnormal sleep/wake cycles and the effect of environmental noise on sleep disruption in the intensive care unit. Am J Respir Crit Care Med. 2001;163(2):451-7.
64. Elliott R, et al. Characterisation of sleep in intensive care using 24-hour polysomnography: an observational study. Crit Care. 2013;17(2):R46.
65. Boyko Y, et al. Sleep quality and circadian rhythm disruption in the intensive care unit: a review. Nat Sci Sleep. 2017;9:277-84.
66. Sanders RD, Maze M. Contribution of sedative-hypnotic agents to delirium via modulation of the sleep pathway. Can J Anaesth. 2011;58(2):149-56.
67. Friese RS. Sleep and recovery from critical illness and injury: a review of theory, current practice, and future directions. Crit Care Med. 2008;36(3):697-705.
68. Opp MR. Sleep and psychoneuroimmunology. Immunol Allergy Clin North Am. 2009;29(2):295-307.
69. Orwelius L, et al. Prevalence of sleep disturbances and long-term reduced health-related quality of life after critical care: a prospective multicenter cohort study. Crit Care. 2008;12(4):R97.

For peer review only - http://bmjopen.bmj.com/site/about/guidelines.xhtml

70. Tembo AC, et al. The experience of sleep deprivation in intensive care patients: findings from a larger hermeneutic phenomenological study. Intensive Crit Care Nurs. 2013;29(6):310-6.
71. Simini B. Patients' perceptions of intensive care. Lancet. 1999;354(9178):571-2.
72. Rotondi AJ, et al. Patients' recollections of stressful experiences while receiving prolonged mechanical ventilation in an intensive care unit. Crit Care Med. 2002;30(4):746-52.
73. Kamdar BB, et al. Sleep deprivation in critical illness: its role in physical and psychological recovery. J Intensive Care Med. 2012;27(2):97-111.
74. Barr J, et al. Clinical practice guidelines for the management of pain, agitation, and delirium in adult patients in the intensive care unit. Crit Care Med. 2013;41(1):263-306.
75. Hetland B, et al. The influence of music during mechanical ventilation and weaning from mechanical ventilation: A review. Heart Lung. 2015;44(5):416-25.
76. Jean R, et al. Effects of deep sedation on sleep in critically ill medical patients on mechanical ventilation. J Sleep Res. 2019:e12894.
77. Pandharipande P, Ely EW. Sedative and analgesic medications: risk factors for delirium and sleep disturbances in the critically ill. Crit Care Clin. 2006;22(2):313-27, vii.
78. Dolan R, et al. A prospective analysis of sleep deprivation and disturbance in surgical patients. Ann Med Surg (Lond). 2016;6:1-5.
79. Sharma MK, et al. Effects of zolpidem on sleep parameters in patients with cirrhosis and sleep disturbances: A randomized, placebo-controlled trial. Clin Mol Hepatol. 2019;25(2):199-209.
80. Huang Y, et al. The effect of zolpidem on sleep quality, stress status, and nondipping hypertension. Sleep Med. 2012;13(3):263-8.
81. Krenk L, et al. Postoperative sleep disturbances after zolpidem treatment in fast-track hip and knee replacement. J Clin Sleep Med. 2014;10(3):321-6.
82. Simons R, et al. Usefulness of temazepam and zaleplon to induce afternoon sleep. Mil Med. 2006;171(10):998-1001.
83. Koelsch S, et al. Adults and children processing music: an fMRI study. Neuroimage. 2005;25(4):1068-76.
84. Menon V, Levitin DJ. The rewards of music listening: response and physiological connectivity of the mesolimbic system. Neuroimage. 2005;28(1):175-84.
85. Koelsch S. A coordinate-based meta-analysis of music-evoked emotions. Neuroimage. 2020;223:117350.
86. Koelsch S. Brain correlates of music-evoked emotions. Nat Rev Neurosci. 2014;15(3):170-80.
87. Chlan L. Effectiveness of a music therapy intervention on relaxation and anxiety for patients receiving ventilatory assistance. Heart Lung. 1998;27(3):169-76.
88. Gillen E, et al. Effects of music listening on adult patients' pre-procedural state anxiety in hospital. JBI Libr Syst Rev. 2008;6(17):686-732.
89. Tracy MF, Chlan L. Nonpharmacological interventions to manage common symptoms in patients receiving mechanical ventilation. Crit Care Nurse. 2011;31(3):19-28.
90. Chanda ML, Levitin DJ. The neurochemistry of music. Trends Cogn Sci. 2013;17(4):179-93.
91. Sigurdardottir GA, et al. A pilot study on high amplitude low frequency-music impulse stimulation as an add-on treatment for depression. Brain Behav. 2019;9(10):e01399.
92. Jain SV, Glauser TA. Effects of epilepsy treatments on sleep architecture and daytime sleepiness: an evidence-based review of objective sleep metrics. Epilepsia. 2014;55(1):26-37.
93. Romero-Osorio O, et al. Changes in sleep patterns after vagus nerve stimulation, deep brain stimulation or epilepsy surgery: Systematic review of the literature. Seizure. 2018;56:4-8.
94. Hoey LM, et al. Sleep assessment of hospitalised patients: a literature review. Int J Nurs Stud. 2014;51(9):1281-8.

For peer review only

For peer review only - http://bmjopen.bmj.com/site/about/guidelines.xhtml

95. Billar RJ, et al. Interventions with Music in PECTus excavatum treatment (IMPECT trial): a study protocol for a randomised controlled trial investigating the clinical effects of perioperative music interventions. BMJ Open. 2020;10(7):e036380.

For peer review only

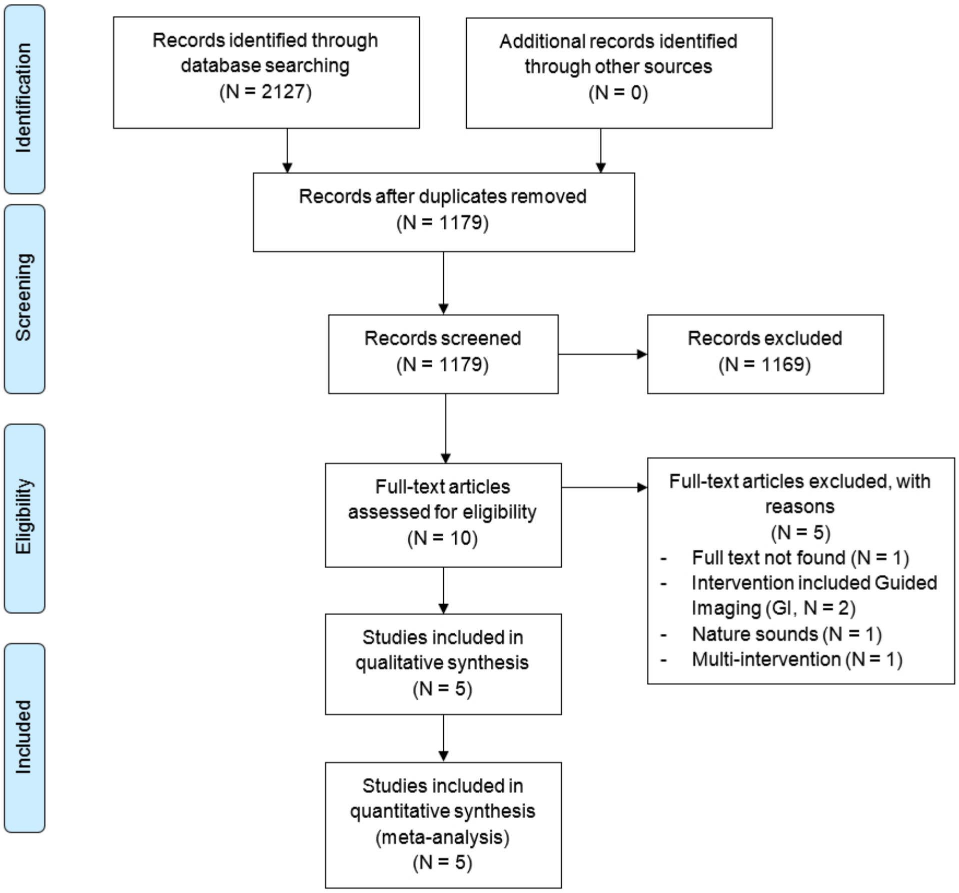

Figure 1. PRISMA Flow Diagram. N indicates the number of articles.

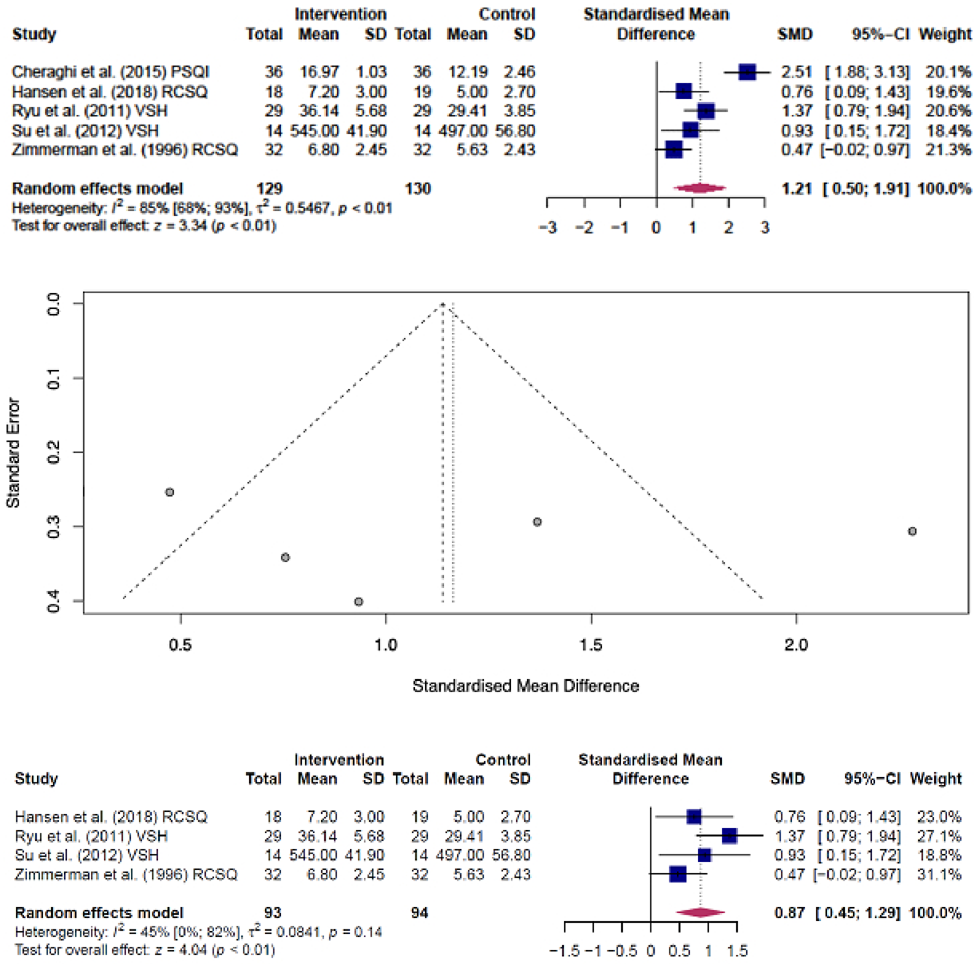

Figure 2A. Forest plot assessing the effect of music on subjective sleep quality. Figure 2B. Funnel plot assessing the effect of music on subjective sleep quality. Figure 2C. Forest plot assessing the effect of music on subjective sleep, excluding the paper of Cheraghi et al.

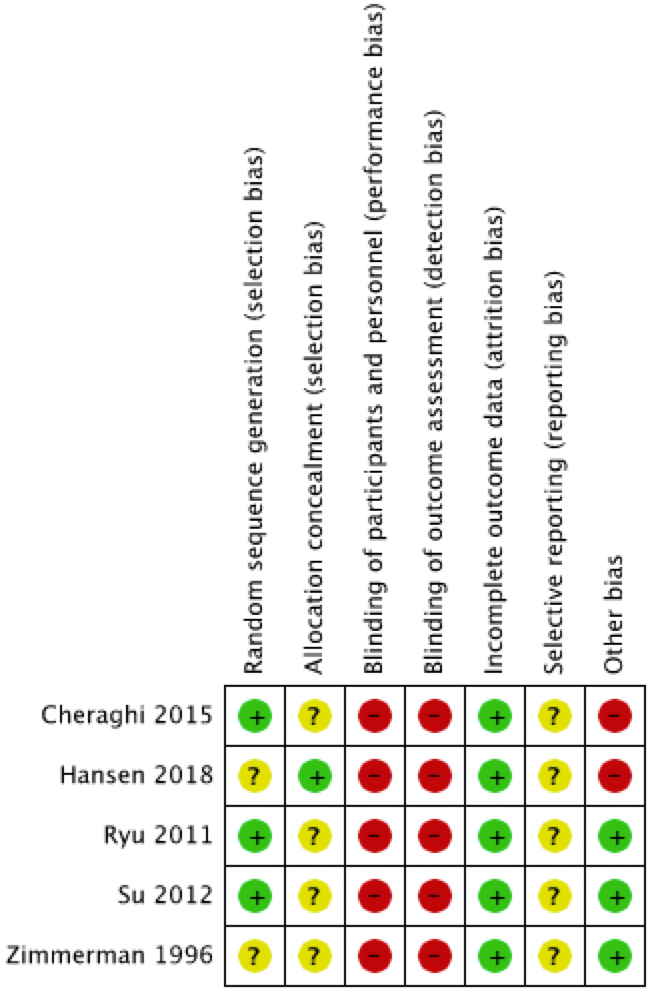

Figure 3. Risk of bias assessment

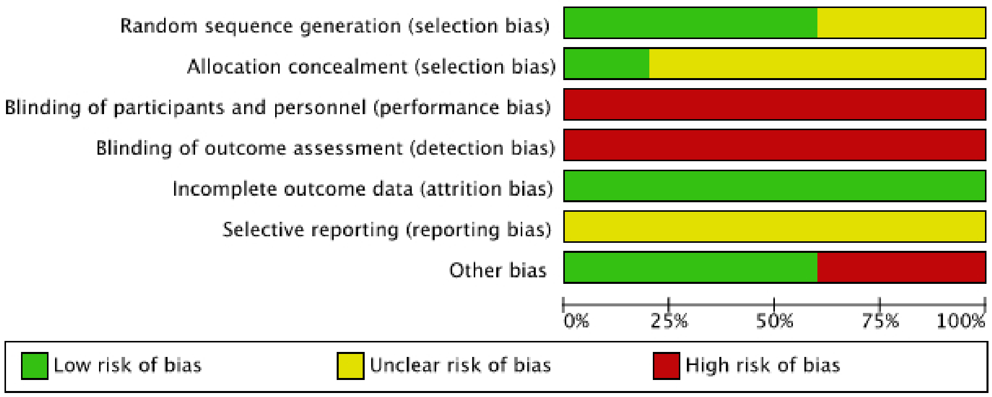

Figure 4. Summary of risk of bias

Supplementary file 1 PRISMA checklist

Section/topic	#	Checklist item	Reported on page #
**TITLE**			
Title	1	Identify the report as a systematic review, meta-analysis, or both.	1
**ABSTRACT**			
Structured summary	2	Provide a structured summary including, as applicable: background; objectives; data sources; study eligibility criteria, participants, and interventions; study appraisal and synthesis methods; results; limitations; conclusions and implications of key findings; systematic review registration number.	2+3
**INTRODUCTION**			
Rationale	3	Describe the rationale for the review in the context of what is already known.	4
Objectives	4	Provide an explicit statement of questions being addressed with reference to participants, interventions, comparisons, outcomes, and study design (PICOS).	5
**METHODS**			
Protocol and registration	5	Indicate if a review protocol exists, if and where it can be accessed (e.g., Web address), and, if available, provide registration information including registration number.	7
Eligibility criteria	6	Specify study characteristics (e.g., PICOS, length of follow-up) and report characteristics (e.g., years considered, language, publication status) used as criteria for eligibility, giving rationale.	7+8
Information sources	7	Describe all information sources (e.g., databases with dates of coverage, contact with study authors to identify additional studies) in the search and date last searched.	7
Search	8	Present full electronic search strategy for at least one database, including any limits used, such that it could be repeated.	Supplementary file 2
Study selection	9	State the process for selecting studies (i.e., screening, eligibility, included in systematic review, and, if applicable, included in the meta-analysis).	7+8
Data collection process	10	Describe method of data extraction from reports (e.g., piloted forms, independently, in duplicate) and any processes for obtaining and confirming data from investigators.	8
Data items	11	List and define all variables for which data were sought (e.g., PICOS, funding sources) and any assumptions and simplifications made.	8

For peer review only - http://bmjopen.bmj.com/site/about/guidelines.xhtml

Risk of bias in individual studies	12	Describe methods used for assessing risk of bias of individual studies (including specification of whether this was done at the study or outcome level), and how this information is to be used in any data synthesis.	9
Summary measures	13	State the principal summary measures (e.g., risk ratio, difference in means).	9
Synthesis of results	14	Describe the methods of handling data and combining results of studies, if done, including measures of consistency (e.g., I^2) for each meta-analysis.	9
Risk of bias across studies	15	Specify any assessment of risk of bias that may affect the cumulative evidence (e.g., publication bias, selective reporting within studies).	9
Additional analyses	16	Describe methods of additional analyses (e.g., sensitivity or subgroup analyses, meta-regression), if done, indicating which were pre-specified.	NA
**RESULTS**			
Study selection	17	Give numbers of studies screened, assessed for eligibility, and included in the review, with reasons for exclusions at each stage, ideally with a flow diagram.	11
Study characteristics	18	For each study, present characteristics for which data were extracted (e.g., study size, PICOS, follow-up period) and provide the citations.	11-15, Tables 1 and 2
Risk of bias within studies	19	Present data on risk of bias of each study and, if available, any outcome level assessment (see item 12).	17
Results of individual studies	20	For all outcomes considered (benefits or harms), present, for each study: (a) simple summary data for each intervention group (b) effect estimates and confidence intervals, ideally with a forest plot.	Figures 2A, B, and C
Synthesis of results	21	Present results of each meta-analysis done, including confidence intervals and measures of consistency.	15-17
Risk of bias across studies	22	Present results of any assessment of risk of bias across studies (see Item 15).	17+18
Additional analysis	23	Give results of additional analyses, if done (e.g., sensitivity or subgroup analyses, meta-regression [see Item 16]).	NA
**DISCUSSION**			
Summary of evidence	24	Summarize the main findings including the strength of evidence for each main outcome; consider their relevance to key groups (e.g., healthcare providers, users, and policy makers).	19
Limitations	25	Discuss limitations at study and outcome level (e.g., risk of bias), and at review-level (e.g., incomplete retrieval of identified research, reporting bias).	21+22
Conclusions	26	Provide a general interpretation of the results in the context of other evidence, and implications for future research.	22+23
**FUNDING**			
Funding	27	Describe sources of funding for the systematic review and other support (e.g., supply of data); role of funders for the systematic review.	23

For peer review only - http://bmjopen.bmj.com/site/about/guidelines.xhtml

Supplementary file 2 Search strategy

Database	Records identified	After de-duplication
embase.com	733	724
Medline Ovid	476	94
Web of science	457	187
Cochrane CENTRAL	261	53
Google scholar	200	121
Total	2127	1179

embase.com

(music/de OR 'music therapy'/de OR (music OR musical OR musicotherap*):ab,ti) AND ('sleep'/de OR 'night sleep'/de OR 'nonREM sleep'/de OR 'REM sleep'/de OR 'sleep induction'/de OR 'sleep pattern'/de OR 'sleep quality'/de OR 'sleep spindle'/de OR 'sleep stage'/de OR 'sleep time'/de OR 'sleep waking cycle'/de OR 'slow wave sleep'/de OR 'sleep disorder'/de OR 'insomnia'/de OR 'sleep parameters'/exp OR 'circadian rhythm'/de OR 'chronobiology'/de OR 'sleep hygiene'/de OR 'sleep deprivation'/de OR 'somnolence'/de OR 'sleep medicine'/exp OR (sleep* OR asleep* OR insomnia OR bedtime OR bed-time OR circadian* OR rem OR nonrem OR chronobiology* *OR somnolence**):ab,ti) NOT ([animals]/lim NOT [humans]/lim) NOT ([Conference Abstract]/lim) AND [English]/lim

Medline Ovid

(music/ OR "music therapy"/ OR (music OR musical OR musicotherap*).ab,ti.) AND (exp sleep/ OR Sleep Wake Disorders/ OR Sleep Deprivation/ OR Circadian Rhythm/ OR (sleep* OR asleep* OR insomnia OR bedtime OR bed-time OR circadian* OR rem OR nonrem OR chronobiology*).ab,ti.) NOT (exp animals/ NOT humans/) AND english.la.

For peer review only - http://bmjopen.bmj.com/site/about/guidelines.xhtml

Web of science

TS=(((music OR musical OR musicotherap*)) AND ((sleep* OR asleep* OR insomnia OR bedtime OR bed-time OR circadian* OR rem OR nonrem OR chronobiology*))) AND DT=(article) AND LA=(english)

Cochrane

((music OR musical OR musicotherap*):ab,ti) AND ((sleep* OR asleep* OR insomnia OR bedtime OR bed-time OR circadian* OR rem OR nonrem OR chronobiology*):ab,ti)

Google scholar

music|musical|musicotherapy sleep|asleep|insomnia|bedtime|"bed-time"|circadian|rem|nonrem|chronobiology

For peer review only

For peer review only - http://bmjopen.bmj.com/site/about/guidelines.xhtml

Supplementary file 3 Sleep questionnaire characteristics

	RCSQ[1]	VSH [2]	PSQI[3]
**Items**	5	15	24
**Item assessment method**	Visual analogue scale	Numeric rating scale	Multiple methods
**Aspects of sleep assessed**	- Sleep depth<br>- Sleep latency<br>- Number of awakening<br>- Efficiency<br>- Sleep quality	- Sleep disturbance (sleep latency, mid-sleep awakenings, soundness of sleep, and movement during sleep)<br>- Effectiveness (rest upon awakening, subjective quality of sleep, and total sleep period)	- Quality<br>- Latency<br>- Duration<br>- Efficiency<br>- Disturbance<br>- Medication<br>- Daytime sleep<br>- Dysfunction
**Timing**	Assess sleep of night before		
**Validated**	Yes	Yes	Yes
**Reliability coefficient**	0.90	0.82	0.70-0.83

PSQI = Pittsburgh sleep quality index; RCSQ = Richards-Campbell sleep questionnaire; VSH = Verran and Snyder-Halpern sleep scale

[1] Richards KC, et al. Measurement of sleep in critically ill patients. J Nurs Meas. 2000;8(2):131-44.
[2] Snyder-Halpern R, Verran JA. Instrumentation to describe subjective sleep characteristics in healthy subjects. Res Nurs Health. 1987;10(3):155-63.
[3] Mollayeva T, et al. The Pittsburgh sleep quality index as a screening tool for sleep dysfunction in clinical and non-clinical samples: A systematic review and meta-analysis. Sleep Med Rev. 2016;25:52-73.

For peer review only - http://bmjopen.bmj.com/site/about/guidelines.xhtml
